# Association of polymorphisms in heat shock protein 70 genes with the susceptibility to noise-induced hearing loss: A meta-analysis

**DOI:** 10.1371/journal.pone.0188195

**Published:** 2017-11-16

**Authors:** Shimin Zong, Xue Zeng, Tianyi Liu, Fangmin Wan, Pan Luo, Hongjun Xiao

**Affiliations:** 1 Department of Otorhinolaryngology, Union Hospital, Tongji Medical College, Huazhong University of Science and Technology, Wuhan, China; 2 Department of Obstetrics and Gynecology, Tongji Hospital, Tongji Medical College, Huazhong University of Science and Technology, Wuhan, China; Huashan Hospital Fudan University, CHINA

## Abstract

**Background:**

Several case-control studies reported the relationship between single nucleotide polymorphisms (SNPs) in HSP70 genes and noise-induced hearing loss (NIHL). However, their conclusions are conflicting. This meta-analysis aims to identify the association of HSP70 variants and NIHL susceptibility.

**Method:**

A systematical literature search was performed in PubMed, Web of Science, EMBASE, and Wanfang Chinese database. The pooled odds radio (OR), 95% confidence interval (CI) and p value were calculated in fixed- or random-effects model according to the I^2^ value in the heterogeneity test.

**Results:**

Four articles containing five studies, including 633 cases and 926 controls, were included. Under the allele, homozygote and dominant model, the pooled ORs (95%CI, p-value) of rs1061581 were 1.32 (1.06–1.67, p = 0.019), 1.93 (1.10–3.36, p = 0.021) and 1.455 (1.408–2.019, p = 0.025), respectively. In addition, a significant association was found between rs2227956 in Caucasians and the NIHL susceptibility under all five genetic models. We did not discover evidence sufficient to prove the associations between the other three SNPs (rs1043618, rs2763979 and rs2075800) and the NIHL susceptibility.

**Conclusion:**

This meta-analysis indicated that the two HSP70 variants, rs1061581 and rs2227956, may serve as genetic susceptibility factors for NIHL. Larger scale studies are required to further update the results.

## Introduction

Noise is one of the most common sources of environmental stress in our contemporary society [1]. Continuous noise or acoustic overstimulation damages the cochlea structure and causes inner ear cell apoptosis, resulting in hearing impairment [2]. Noise-induced hearing loss (NIHL) has been the most recorded occupational disorder in the world, accounting for 7 to 21% of hearing loss [3, 4]. However, once NIHL occurs, few therapeutic methods would be clinically effective to date [5]. Thus, to establish a practical prediction system to individual NIHL susceptibility is necessary.

It cannot be ignored that the susceptibility to NIHL among individuals is obviously diverse; some individuals are more susceptible to NIHL than others. Several studies have suggested that this individual difference in susceptibility to NIHL is due to the diverse genetic background among individuals [6]. The genotypes or single nucleotide polymorphisms (SNPs) of some genes have been demonstrated to be related to individual NIHL susceptibility, such as *SOD2* [7], *GST* [8], *PCDH1*5 and *MYH14* [9], but these genetic variations still cannot explain all individual differences. Therefore, it is necessary to identify more NIHL-associated genes or genetic polymorphisms to further improve the genetic predictive system of NIHL.

Recent studies demonstrated that the disturbance of cellular proteostasis is critical in the development of NIHL [10]. The heat shock protein 70kD (HSP70) family, as molecular chaperones, are important for protein folding, modification, maturing and cellular normal function [11]. An increased expression of HSP70 has been found in cochlea cells under noise exposure and they further act as a protective factor in the development of NIHL [12]. What is noteworthy is that some studies indicated a close association between several SNPs in HSP70 genes, including rs1043618 [13, 14], rs2763979 [15], rs2227956 [14, 15], and rs1061581 [14] (the characteristics of these SNPs are listed in Table 1), and NIHL susceptibility, whereas no positive result for rs1043618, rs2227956 and rs1061581 was found in Yang‘s study [16]. For each SNP site, the conclusion in different studies is also diverse. A reason for the conflictive results is the heterogeneity among and the limitations in these studies, such as ethnicity, sample size, study design and many other factors, while meta-analysis has an advantage in assessing the heterogeneity and overcoming the limitations.

**Table 1:** Characteristics of the five SNPs in HSP70 genes.

SNP	Location (gene)	Location (chromosome)	Synonymous or not	Variation in protein level
rs1043618	the 5’ UTR of *HSPA1A*	6p21.3	-	NA
rs2763979	936 bp upstream of the 5’ end of the HSPA1B*	6p21.3	-	NA
rs2227956	*HSPA1A*	6p21.3	a nonsynonymous variation	changes methionine to threonine resulting in the 3D structure alteration
rs2075800	*HSPA1A*	6p21.3	a nonsynonymous variation	changes glutamic acid to lysine resulting in the 3D structure alteration
rs1061581	*HSPA1B*	6p21.3	a synonymous variation	-

UTR: untranslated region; NA: not available;

* be considered belonging to the *HSPA1B*.

There is no meta-analysis or genome-wide association studies (GWAS) on the association of HSP70 polymorphisms with NIHL susceptibility to date. Aiming at evaluating the potential value of HSP70 variants in prediction for individual NIHL susceptibility, we focus on five reported SNPs in HSP70 genes here, perform a meta-analysis to address these conflicting results and assess whether HSP70 polymorphisms are associated with the susceptibility to NIHL. The results of this meta-analysis will provide theoretical basis for the application of the SNPs in HSP70 genes in the individualized prevention system to NIHL.

## Methods

### Search strategy

A comprehensive literature search was performed in the following English and Chinese databases: (1) PubMed; (2) Web of Science; (3) EMBASE; and (4) Wanfang Chinese database. The MeSH and free terms were all included in our search terms, which are listed as follows: “heat shock protein 70”, “hsp70”, “noise”, “hearing loss”, “noise-induced hearing loss” and “NIHL”. Our search logic in the PubMed database is listed as follows: “((heat shock protein 70) OR (hsp70)) AND ((noise AND (hearing loss) OR (noise-induced hearing loss) OR (hearing loss, noise-induced [MeSH]) OR NIHL)”. The publication languages were limited to English and Chinese. All studies that we searched were published before April 20^th^, 2017. We also manually checked all articles listed in the reference lists of the retrieved literatures.

### Inclusion criteria

All studies included in our meta-analysis need to be confirmed with the following criteria: (1) independent case-control studies investigating the relationship between the SNPs in HSP70 genes and the development of NIHL; (2) studies including sufficient and definite original data (the genotype frequency of each SNP in HSP70 genes in the case and control groups) in which the odds ratio (OR) with its 95% confidential interval (CI) of each genotype at every SNP site can be calculated; (3) two independent sample sets in one study were considered as two different studies; and (4) the data in the latest publication were used when duplicate publications were found.

### Data extraction strategy and quality assessment

Data in the included studies were independently extracted by three authors (S Zong, X Zeng and T Liu) with the same “Data Extraction Form”. The following information was extracted from every included study: the mutation site of each SNP, first author’s last name, publication year, country, ethnicity, workplace of each sample set, standard of noise exposure, diagnosis criteria of hearing impairment or NIHL susceptibility, numbers of cases and controls, and consistency with Hardy-Weinberg Equilibrium (HWE) in the control group. The linkage disequilibrium (LD) pattern (r^2^ value and D’ value) between SNPs in different populations was extracted from the SNAP website (http://www.broadinstitute.org/mpg/snap) based on the data from the 1000 Genome Project.

The Newcastle-Ottawa scale (NOS) was used to assess the quality of each included study. The studies with a score ≥ 7 were considered high-quality studies. When there is conflict in the process of study selection, data extraction or quality assessment, the reviewers discussed all items until they reached consensus.

### Meta-analysis

SNPs reported in two or more studies were evaluated in a meta-analysis. The association between SNPs in the HSP70 genes and NIHL susceptibility was tested by pooled OR and 95%CI. The allele (A vs. B), homozygote (AA vs. BB), heterozygote (AA vs. AB), dominant (AA vs. AB + BB) and recessive (AA + AB vs. BB) model were all applied for genotype comparison to minimize the possibility of the second type of error. The heterogeneity among the included studies was evaluated by the Q and I^2^ tests. The pooled ORs and 95%CIs were calculated under the fixed-effects model for p > 0.1 and I^2^ < 50%; otherwise, under the random-effects model with I^2^ ≥ 50%. Woolf’s method was applied to estimate the 95%CIs. The Z-test was applied to identify the association between the SNPs in HSP70 genes and NIHL susceptibility. We considered there was statistical significance when the overall 95% CI did not include 1 and the p-value transformed from the Z score was less than 0.05. Sub-group analysis based on ethnicity, study quality and accordance with HWE in the controls was performed. Sensitivity analysis was applied to estimate the stability of the pooled results. Publication bias for rs1043618, rs1061581 and rs2227956 were evaluated by funnel plots and the Egger’s test. For p<0.05 or a 95%CI that did not contain 0 in the Egger’s test, publication bias was considered present. HWE of the genotype distribution in the control group was evaluated by the Pearson’s X^2^ test. All statistical analysis was performed by Stata 13.1 software.

## Results

### Literature search and characteristics of included studies

**Fig 1:**
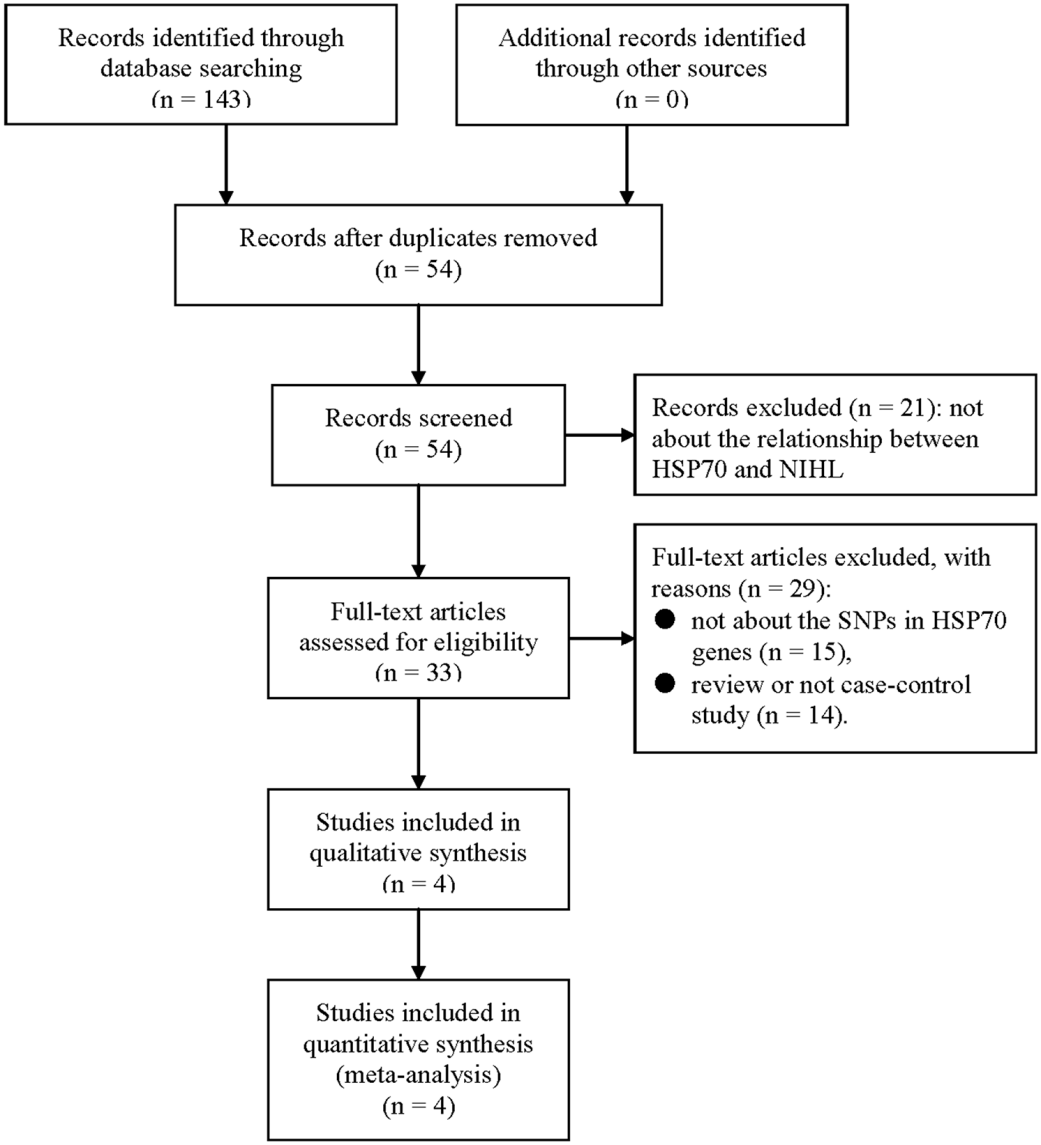
Flow diagram of study selection process.

Our literature selection process is presented in Fig 1. One hundred and forty-three articles were found after our primary literature search. Fifty-four articles remained for screening the title and abstract after removing repetitive records. Twenty-one articles were excluded because the studies were not about the relationship between HSP70 and NIHL. The remaining 33 articles were assessed for eligibility via full-text screening. Twenty-nine articles were further excluded because they are reviews (n = 14) or not about the SNPs in HSP70 genes (n = 15). Finally, four articles [13–16] on 5 independent studies were included in the quality assessment and meta-analysis. Therefore, a total of 633 cases with NIHL and 926 controls were included. Table 2 summarized the basic information, including the NOS scores of the 5 eligible studies. Table 3 presents the genotype distribution of SNPs in HSP70 genes in the 5 eligible studies.

**Table 2:** Characteristics of included studies.

First author (year)	Country	Ethnicity	Workplace	Sample size (M/F)		Age		Genotype method	NOS score
				Case	Control	Case	Control		
Li (2017)	China (mainland)	Asian (Chinese)	Steel factory	286(274/12)	286(274/12)	45.5 ± 8.1	39.8 ± 8.1	PCR(SNP scan^™^)	7
Chang (2011)	China (Taiwan)	Asian (Chinese)	Factories not clearly showed	27(27/10)	322(309/13)	45.15 ± 6.74	44.05 ± 7.82	Real time—PCR	8
Konings (2009)	Sweden	Caucasian (Swedish)	2 paper pulp mills and 1 steel factory	108(NA)	98(NA)	NA	NA	PCR(SNaPshot^™^)	6
Konings (2009)	Poland	Caucasian (Polish)	Different factories	119(NA)	119(NA)	NA	NA	PCR(SNaPshot^™^)	7
Yang (2009)	China (mainland)	Asian (Chinese)	Motor factory	93(77/16)	101(55/46)	35.2 ± 6.9	33.0 ± 6.1	PCR	7

M/F: male/female. NOS: Newcastle-Ottawa Scale. PCR: polymerase chain reaction. NA: not available. TM: trademark.

**Table 3:** Genotype distribution of HSP70 SNPs.

SNP	First author (year)	Case				Control				HWE	
		AA	AB	BB	Total	AA	AB	BB	Total	X^2^	P
**rs1043618**	Li (2017)	124	117	45	286	130	125	31	286	0.01352	0.907435
(G> C)	Chang (2011)	8	18	1	27	153	139	30	322	0.037921	0.845602
	Konings (2009, Swedish)	31	51	11	93	49	44	7	100	0.468949	0.493471
	Konings (2009, Polish)	44	58	14	116	46	58	12	116	0.009615	0.92189
	Yang (2009)	37	43	13	93	35	48	18	101	0.047963	0.826647
**rs2075800**	Li (2017)	128	128	30	286	112	132	42	286	0.093721	0.759499
(C > T)	Chang (2011)	10	15	2	27	113	166	43	322	2.175678	0.140208
**rs2227956**	Li (2017)	204	64	8	276	201	73	2	276	2.856668	0.090996
(A > G)	Konings (2009, Swedish)	64	27	0	91	54	39	5	98	0.367347	0.544454
	Konings (2009, Polish)	95	22	1	118	81	32	4	117	0.143747	0.704584
	Yang (2009)	58	34	1	93	67	32	2	101	0.673503	0.411833
**rs2763979**	Li (2017)	104	133	49	286	116	139	31	286	1.253581	0.26287
(C > T)	Chang (2011)	18	9	0	27	179	124	19	322	0.165829	0.683846
**rs1061581**	Konings (2009, Swedish)	24	55	13	92	44	45	11	100	0.009975	0.920442
(A > G)	Konings (2009, Polish)	37	61	18	116	43	56	15	114	0.236309	0.626885
	Yang (2009)	43	41	9	93	50	48	3	101	4.591447	**0.032132**^#^

HWE: Hardy-Weinberg equilibrium.

^#^ P < 0.05, showing statistically significant difference.

### The association between SNPs in HSP70 genes and NIHL susceptibility

**Fig 2:**
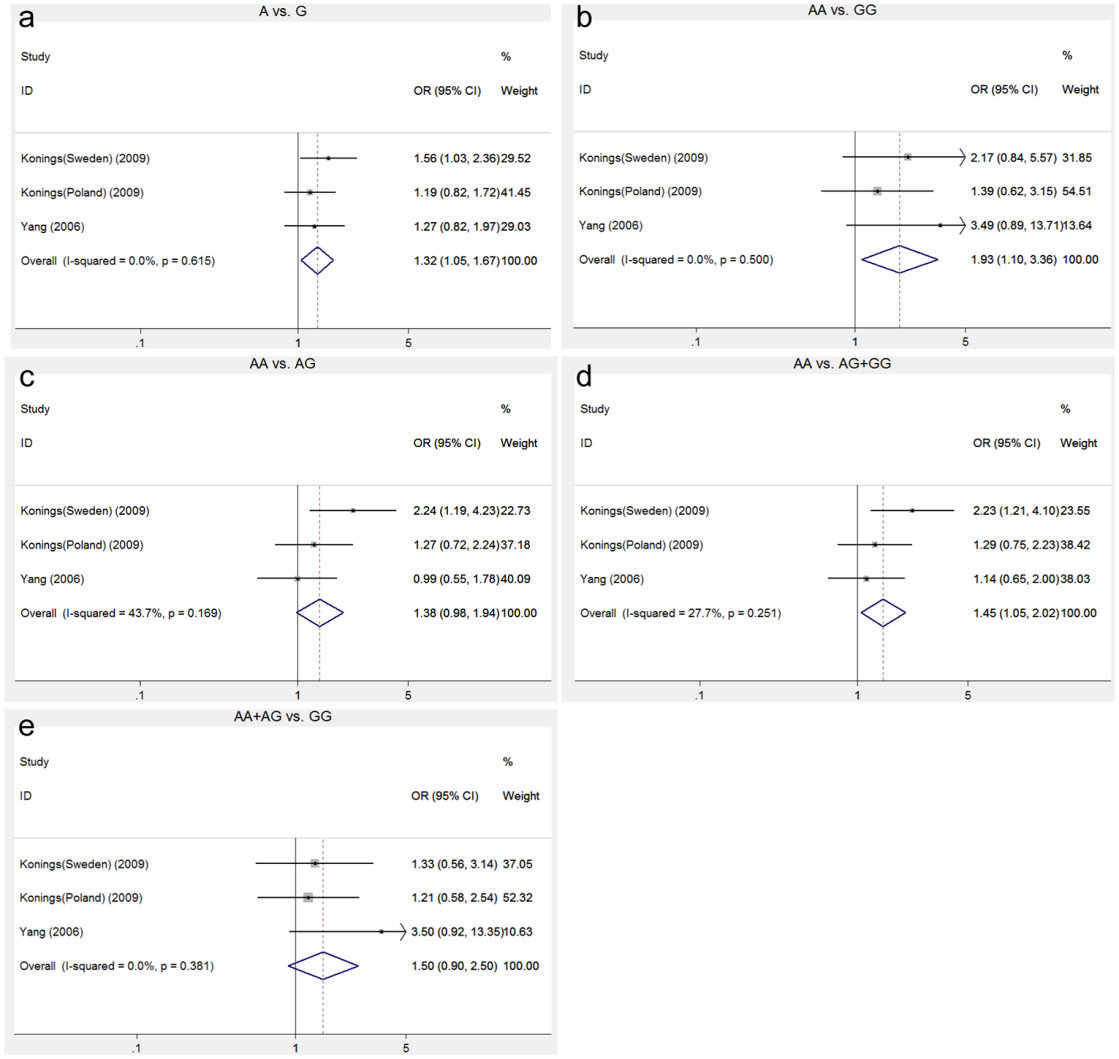
The association between rs1061581 and NIHL susceptibility in different genetic models.

We selected five SNPs (rs1061581, rs1043618, rs2227956, rs2075800 and rs2763979) in HSP70 genes for the meta-analysis. Five genetic models were used for the genotype comparison. For SNP rs1061581, two studies on Caucasian populations and one study on Asian populations containing 301 cases and 315 controls were included. The pooled ORs and 95% CIs were 1.32 (1.06–1.67, p = 0.019), 1.93 (1.10–3.36, p = 0.021) and 1.455 (1.408–2.019, p = 0.025) in the allele, homozygous and dominant model, respectively (Fig 2 and Table 4). These results indicated that there is a close association between the SNP rs1061581 in the *HSPA1B* gene and NIHL susceptibility. For rs2227956, the pooled ORs and 95% CIs in Caucasian were 0.535 (0.368–0.779, p = 0.001), 0.135 (0.024–0.764, p = 0.024), 0.585 (0.379–0.903, p = 0.016), 0.531 (0.374–0.812, p = 0.004) and 0.157 (0.028–0.889, p = 0.036) in the allele, homozygote, heterozygote, dominant and recessive models, respectively (Table 4 and S1 Fig). For rs1043618, rs2763979 and rs2075800, no significant association was found between the SNPs and NIHL susceptibility in any of the five genetic models for the Asian and the Caucasian populations, respectively, or across the ethnicity. The details of the overall analysis of the association of the five SNPs in HSP70 genes and NIHL susceptibility are listed in Table 4.

**Table 4:** Overall analysis of the association between HSP70 SNPs and NIHL susceptibility.

SNP	Ethnicity	Study number	Case/Control	Genetic model	I^2^ (%)	Model	OR (95% CI)	Z score	P (Z)
**rs1043618**	**Mixed population**	5	615/925	G vs. C	19.7	F	1.150 (0.978, 1.351)	1.70	0.090
(G > C)				GG vs. CC	10.6	F	1.301 (0.909, 1.863)	1.44	0.151
				GG vs. GC	44.0	F	1.151 (0.912, 1.454)	1.18	0.236
				GG vs. GC+CC	39.5	F	1.188 (0.952, 1.484)	1.53	0.127
				GG+GC vs. CC	5.1	F	1.228 (0.879, 1.716)	1.21	0.228
	**Asian subgroup**	3	406/709	G vs. C	18.2	F	1.092 (0.897, 1.330)	0.88	0.381
				GG vs. CC	29.4	F	1.086 (0.594, 1.987)	0.27	0.789
				GG vs. GC	54.5	R	1.144 (0.695, 1.885)	0.53	0.597
				GG vs. GC+CC	42.6	F	1.099 (0.839, 1.440)	0.68	0.494
				GG+GC vs. CC	45.4	F	1.165 (0.784, 1.729)	0.76	0.450
	**Caucasian subgroup**	2	209/216	G vs. C	42.9	F	1.278 (0.964, 1.694)	1.70	0.088
				GG vs. CC	4.0	F	1.673 (0.824, 3.253)	1.41	0.159
				GG vs. GC	44.7	F	1.349 (0.900, 2.023)	1.45	0.147
				GG vs. GC+CC	52.2	R	1.418 (0.803, 2.503)	1.20	0.228
				GG+GC vs. CC	0.0	F	1.403 (0.784, 2.633)	1.06	0.291
	**High quality**	4	525/828	G vs. C	0.0	F	1.089(0.914, 1.297)	0.96	0.339
	**subgroup**			GG vs. CC	0.0	F	1.191(0.812, 1.748)	0.89	0.371
				GG vs. GC	31.8	F	1.060(0.823, 1.366)	0.45	0.653
				GG vs. GC+CC	14.0	F	1.094(0.860, 1.391)	0.73	0.464
				GG+GC vs. CC	18.0	F	1.169(0.819, 1.669)	0.86	0.389
**rs2227956**	**Mixed population**	4	578/592	A vs. G	63.7	R	0.780 (0.528, 1.847)	1.25	0.213
(A>G)				AA vs. GG	62.4	R	0.549 (0.090, 3.348)	0.65	0.516
				AA vs. AG	27.1	F	0.802 (0.619, 1.040)	1.67	0.096
				AA vs. AG + GG	50.7	R	0.772 (0.529, 1.127)	1.34	0.180
				AA + AG vs. GG	60.3	R	0.593 (0.013, 3.425)	0.58	0.550
	**Asian subgroup**	2	369/377	A vs. G	0.0	F	1.064 (0.802, 1.410)	0.43	0.668
				AA vs. GG	41.3	F	2.328 (0.071, 7.727)	1.38	0.168
				AA vs. AG	0.0	F	0.959 (0.693, 1.327)	0.25	0.800
				AA vs. AG + GG	0.0	F	1.011 (0.734, 1.389)	0.07	0.945
				AA + AG vs. GG	47.7	F	2.335 (0.711, 7.671)	1.40	0.162
	**Caucasian subgroup**	2	209/215	**A vs. G**	0.0	F	**0.535 (0.368, 0.779)**	3.26	**0.001**
				**AA vs. GG**	0.0	F	**0.135 (0.024, 0.764)**	2.26	**0.024**
				**AA vs. AG**	0.0	F	**0.585 (0.379, 0.903)**	2.42	**0.016**
				**AA vs. AG + GG**	0.0	F	**0.531 (0.374, 0.812)**	2.92	**0.004**
				**AA + AG vs. GG**	0.0	F	**0.157 (0.028, 0.889)**	2.09	**0.036**
	**High quality**	3	470/494	A vs. G	55.9	R	0.886(0.595, 1.319)	0.59	0.552
	**subgroup**			AA vs. GG	59.4	R	0.915(0.144, 5.827)	0.09	0.925
				AA vs. AG	29.5	F	0.861(0.646, 1.147)	1.02	0.307
				AA vs. AG + GG	44.3	F	0.882(0.667, 1.166)	0.88	0.377
				AA + AG vs. GG	58.5	R	0.950(0.153, 5.901)	0.06	0.956
**rs1061581**	**Mixed population**	3	301/315	**A vs. G**	0.0	F	**1.322 (1.046, 1.671)**	2.34	**0.019**
(A>G)				**AA vs. GG**	0.0	F	**1.926 (1.104, 3.359)**	2.31	**0.021**
				AA vs. AG	43.7	F	1.378 (0.981,1.937)	1.85	0.065
				**AA vs. AG + GG**	27.7	F	**1.455 (1.408, 2.019)**	2.24	**0.025**
				AA + AG vs. GG	0.0	F	1.500 (0.901, 2.496)	1.56	0.119
	**Caucasian subgroup**	2	251/264	**A vs. G**	0.0	F	**1.342 (1.017, 1.771)**	2.08	**0.037**
	(HWE P > 0.05			AA vs. GG	0.0	F	1.679 (0.907, 3.109)	1.65	0.099
	subgroup)			**AA vs. AG**	41.9	F	**1.636 (1.073, 2.493)**	2.29	**0.022**
				**AA vs. AG + GG**	41.0	F	**1.648 (1.100, 2.468)**	2.42	**0.015**
				AA + AG vs. GG	0.0	F	1.262 (0.720, 2.210)	0.81	0.416
	**High quality**	2	107/217	A vs. G	0.0	F	1.222(0.920, 1.624)	1.38	0.167
	**subgroup**			AA vs. GG	21.7	F	1.814(0.912, 3.606)	1.70	0.090
				AA vs. AG	0.0	F	1.124(0.748, 1.689)	0.56	0.572
				AA vs. AG + GG	0.0	F	1.217(0.823, 1.800)	0.98	0.326
				AA + AG vs. GG	46.0	F	1.599(0.848, 3.014)	1.45	0.147
**rs2075800**	**Asian**	2	313/608	C vs. T	0.0	F	0.182 (0.684, 1.017)	1.82	0.069
(C>T)				CC vs. TT	0.0	F	0.612 (0.370, 1.013)	1.91	0.056
				CC vs. CT	0.0	F	0.872 (0.631, 1.206)	0.83	0.408
				CC vs. CT + TT	0.0	F	0.811 (0.596, 1.103)	1.33	0.182
				CC + CT vs. TT	0.0	F	0.658 (0.410, 1.055)	1.74	0.082
**rs2763979**	**Asian**	2	313/608	C vs. T	71.7	R	0.939 (0.462, 1.909)	0.17	0.862
(C>T)				CC vs. TT	44.6	F	1.561 (0.850, 2.564)	1.76	0.079
				CC vs. CT	0.0	F	1.003 (0.724, 1.389)	0.02	0.988
				CC vs. CT + TT	50.0	R	0.971 (0.537, 1.753)	0.10	0.921
				CC + CT vs. TT	34.4	F	1.550 (0.975, 2.464)	1.85	0.064

F: fixed-effects model. R: random-effects model. P< 0.05, showing statistically significant difference.

### Sensitivity analysis

We estimated the stability of the pooled ORs of the SNPs (rs1043618, rs1061581 and rs2227956) by eliminating each of the included studies in turn. For the three SNPs mentioned above, no significant alteration of the pooled ORs was found as any of the included studies was omitted (S2 Fig). These results indicate that the corresponding ORs were relatively robust. For rs2075800 and 2763979, we did not perform the sensitivity analysis because of the limited number of included studies.

### Publication bias

The funnel plots looked symmetric for three SNPs (rs1043618, rs1061581 and rs2227956) in all five genetic models. We did not test the publication bias for rs2075800 and rs2763979 because of the limited number of included studies. Additionally, we did not found any publication bias via Egger’s test (S1 Table).

## Discussion

NIHL affects the life quality of millions of people worldwide [17, 18], especially industrial workers [14]. What cannot be ignored is that the genetic background has a close association with the individual NIHL susceptibility. Therefore, it is essential to identify more genetic markers that can predict individual NIHL susceptibility potentially. The abnormity of HSP70 have been demonstrated to be related to many diseases, such as obesity [19], congenital sideroblastic anemia [20], coronary artery disease [21] and age-related cataract [22]. In the research field of NIHL, two studies both suggested an important role of HSP70 in the resistance to NIHL [12, 23]. Moreover, the participation of HSP70 in the alleviation effect of dexamethasone on NIHL was also indicated [24]. In addition, several SNPs in HSP70 genes are further considered relating to NIHL susceptibility, but the results are inconsistent [13–16]. Therefore, to comprehensively and reliably understand the association between SNPs in HSP70 genes and NIHL susceptibility, we performed this meta-analysis that includes larger samples of 633 cases with NIHL and 926 controls.

A significant relationship was seen between rs1061581 and NIHL susceptibility (Table 4 and Fig 2), which indicated that the *G* allele is a potential risk factor to NIHL susceptibility. Rs1061581 has been considered a major and synonymous SNP in *HSPA1B* gene, which is located at chromosome 6p21.3. Its protein product, HSP-2, is 641 amino acids in length and stress-inducible [25]. However, the way in which rs1061581 variant affected the HSP-2 protein remains unclear, which needs to be further explored. The association of rs2227956 with NIHL is statistically significant only in the Caucasian population (Table 4 and S1 Fig). This phenomenon is mostly due to the heterogeneity in the genetic background of different ethnicities. Rs2227956 in *HSPA1A* is also located at chromosome 6p21.3. A variation from *A* to *G* changes methionine to threonine resulting in the 3D structure alteration of HSP-1, which may affects its function as a molecular chaperone [25]. We found no association between rs1043618, rs2763979 and rs2075800 and NIHL susceptibility.

Heterogeneity is a major problem that affects the reliability of the pooled ORs in meta-analysis. In our meta-analysis, mild to moderate heterogeneity was observed in most SNPs (Table 4). The heterogeneity in our meta-analysis could be explained by the following features. (1) Individuals from different ethnicities have diverse genetic background, such as different LD patterns and different minor allele frequency (MAF) of these SNPs among the population (S2 Table). (2) The diagnostic criteria of hearing loss or NIHL susceptibility in different studies are not in full accord. For example, the frequency in which hearing thresholds were examined differs from study to study. Although the diagnostic criteria in each study were well-founded, there was heterogeneity from these differences. (3) The quality of the included studies is diverse. We used the NOS scale to evaluate the quality of these studies and found the quality of the included studies varied (Table 2), which may be a potential source of heterogeneity.

There are several limitations in this meta-analysis. (1) We only searched the articles published in English and Chinese. The articles in other languages were not included in this meta-analysis, which may increase the publication bias. (2) The number of included studies is relatively low. For rs2075800 and rs2763979, only two studies were included in this meta-analysis. Their funnel plots were very asymmetric, which indicated there is significant publication bias. (3) The sample size in some included studies was relatively small, which may increase the heterogeneity. Although 633 cases and 926 controls are included in this meta-analysis, the conclusion must be updated further by including larger sample reports.

To the best of our knowledge, this is the first meta-analysis on the association between the SNPs in HSP70 genes and NIHL susceptibility. Our results indicated that the allele *G* in rs1061581 and rs2227956 (only in Caucasians) may serve as genetic susceptibility factors for NIHL. However, more studies with larger sample sets from different ethnicities are needed to further confirm the relationship between HSP70 polymorphisms and NIHL susceptibility due to the limitations in this meta-analysis.

## Supporting information

S1 Diagram PRISMA 2009 flow diagram. (DOC)

S1 Appendix List of excluded studies with reasons. (DOCX)

S1 Table Publication bias analyses (Egger's test). (DOCX)

S2 Table The MAF and LD pattern of the four investigated SNPs in HSP70 genes in different populations. (DOCX)

S1 Checklist PRISMA 2009 checklist. (DOC)

S2 Checklist Metaanalysison geneticassociationstudies checklist. (DOCX)

S1 Fig The association between rs2227956 and NIHL susceptibility in different genetic models. (TIF)

S2 Fig Sensitivity analysis of the pooled effective size on the association between rs1061581, rs2227956 and rs1043618 and NIHL susceptibility in different genetic models. (TIF)
