# Correction: Association of polymorphisms in heat shock protein 70 genes with the susceptibility to noise-induced hearing loss: A meta-analysis

**DOI:** 10.1371/journal.pone.0242647

**Published:** 2020-11-17

**Authors:** The PLOS ONE Editors

Questions have been raised about similarities between this article [1] and a closely related meta-analysis [2]. The authors confirmed that the work reported in [1] was completed independently of the other group, and that they were unaware of the related work [2] prior to its publication.

In following up on the above issue, *PLOS ONE* reassessed the article together with a member of the journal’s Editorial Board and a statistical reviewer. The concerns raised in this post-publication assessment and the information and reanalyses provided by the authors in response are outlined below.

1. Concerns were raised about the statistical model used in the published meta-analysis [1] and the rationale provided for the choice of model. It was also noted that there are some discrepancies between results reported in Table 4 and Figure S1 in [1], in instances where Table 4 reports results from the random effects model and the corresponding forest plot in S1 Fig presents results from the fixed effect model.
  The authors commented that the selection of effect model was made according to results of the heterogeneity test. The statistical reviewer advised that the decision as to what model is used should be based on the assumptions underlying the different models, and that this decision should be made before the beginning of the study and should not be influenced by the results.
  In response to the post-publication assessment, the authors commented that due to the small number of included studies it is difficult to estimate the variance between studies which could lead to an incorrect application of the random effects model. They reanalysed the data using a fixed-effects model; the results of the reanalyses are provided here in the updated version of Table 4 and in S3 and S4 Files.
2. Funnel plots were not shown in [1] and are included with this notice in S2 File. Concerns were raised about the validity and/or reliability of funnel plots and Egger’s tests for evaluating publication bias in this study, as these methods are not recommended for meta-analyses involving <10 studies [3]. In light of this issue, the results of the publication bias analyses in S1 Table of [1] should be disregarded or interpreted with caution; conclusions cannot be drawn as to the existence of publication bias.
  The authors commented that although publication bias may exist in this dataset the results of sensitivity analyses indicated that the combination of effect sizes is reliable.
3. The direction of effects was not correctly reported in the discussion of odds ratio (OR) and confidence interval (CI) results in the “The association between SNPs in HSP70 genes and NIHL susceptibility” section. The authors provided the following corrections and clarifications:
  - For rs22227956, the pooled ORs<1 and CIs of OR do not contain the value 1 in Caucasian subgroup, which means the G allele in Caucasian individuals was associated with increased resistance to NIHL.
  - For rs1061581, the pooled ORs>1 and CIs of OR do not contain the value 1 in Caucasian subgroup and mixed population under the allele model, heterozygote model, and dominant model, which means that the G allele in individuals (and especially Caucasian individuals) was associated with increased susceptibility to NIHL.
4. The statistical reviewer raised concerns about the interpretation of the results and noted that non-significant differences should not be interpreted as evidence of no effect. In light of this, the last sentence of second paragraph in the discussion section is updated to: “We found no statistically significant association between rs1043618, rs2763979 and rs2075800 and NIHL susceptibility.”
5. The Yang et al. (2006) article was incorrectly referenced as “Yang (2009)” in Tables 2 and 3, and the Hardy-Weinberg Equilibrium results were misreported in Table 3 for the Konings (2009, Polish) study. These errors are addressed in updated versions of Tables 2 and 3 provided with this notice.
6. The title of the second table in S2 Table of [1] was incorrect and is updated to: “The MAF of the four investigated SNPs in HSP70 genes from different populations.” A revised version of S2 Table is provided here.
7. The title of S1 Fig in [1] did not accurately reflect the figure contents. To address this, the S1 Fig title is updated to, “The forest plots of subgroup analysis based on the ethnicity and the association between rs2227956 and NIHL susceptibility in different genetic models.”
8. Questions were asked about the availability of data underlying this study. The “workplace of each sample set” and “linkage disequilibrium (LD) pattern (r2 value and D’ value) between SNPs in different populations” data extracted for the meta-analysis are reported in Table 2 and S2 Table in [1]. Additional data extracted for this study are in S5 File with this notice. The authors did not respond to the journal’s request for the full list of included articles or the full list of excluded articles with reasons for exclusions.

**Table 2:** Characteristics of included studies.

First author (year)	Country	Ethnicity	Workplace	Sample size (M/F)		Age		Genotype method	NOS score
				Case	Control	Case	Control		
Li (2017)	China (mainland)	Asian (Chinese)	Steel factory	286(274/12)	286(274/12)	45.5 ± 8.1	39.8 ± 8.1	PCR (SNP scan^™^)	7
Chang (2011)	China (Taiwan)	Asian (Chinese)	Factories not clearly showed	27(27/10)	322(309/13)	45.15 ± 6.74	44.05 ± 7.82	Real time—PCR	8
Konings (2009)	Sweden	Caucasian (Swedish)	2 paper pulp mills and 1 steel factory	108(NA)	98(NA)	NA	NA	PCR(SNaPshot^™^)	6
Konings (2009)	Poland	Caucasian (Polish)	Different factories	119(NA)	119(NA)	NA	NA	PCR(SNaPshot^™^)	7
Yang (2006)	China (mainland)	Asian (Chinese)	Motor factory	93(77/16)	101(55/46)	35.2 ± 6.9	33.0 ± 6.1	PCR	7

M/F: male/female. NOS: Newcastle-Ottawa Scale. PCR: polymerase chain reaction. NA: not available. TM: trademark.

**Table 3:** Genotype distribution of HSP70 SNPs.

SNP	First author (year)	Case				Control				HWE	
		AA	AB	BB	Total	AA	AB	BB	Total	X^2^	P
**rs1043618**	Li (2017)	124	117	45	286	130	125	31	286	0.01352	0.907435
(G > C)	Chang (2011)	8	18	1	27	153	139	30	322	0.037921	0.845602
	Konings (2009, Swedish)	31	51	11	93	49	44	7	100	0.468949	0.493471
	Konings (2009, Polish)	44	58	14	116	46	58	12	116	1.024621	0.31443
	Yang (2006)	37	43	13	93	35	48	18	101	0.047963	0.826647
**rs2075800**	Li (2017)	128	128	30	286	112	132	42	286	0.093721	0.759499
(C > T)	Chang (2011)	10	15	2	27	113	166	43	322	2.175678	0.140208
**rs2227956**	Li (2017)	204	64	8	276	201	73	2	276	2.856668	0.090996
(A > G)	Konings (2009, Swedish)	64	27	0	91	54	39	5	98	0.367347	0.544454
	Konings (2009, Polish)	95	22	1	118	81	32	4	117	0.143747	0.704584
	Yang (2006)	58	34	1	93	67	32	2	101	0.673503	0.411833
**rs2763979**	Li (2017)	104	133	49	286	116	139	31	286	1.253581	0.26287
(C > T)	Chang (2011)	18	9	0	27	179	124	19	322	0.165829	0.683846
**rs1061581**	Konings (2009, Swedish)	24	55	13	92	44	45	11	100	0.009975	0.920442
(A > G)	Konings (2009, Polish)	37	61	18	116	43	56	15	114	0.236309	0.626885
	Yang (2006)	43	41	9	93	50	48	3	101	4.591447	**0.032132**^#^

HWE: Hardy-Weinberg equilibrium.

^#^ P < 0.05, showing statistically significant difference.

**Table 4:** Overall analysis of the association between HSP70 SNPs and NIHL susceptibility.

SNP	Ethnicity	Study number	Case/Control	Genetic model	I^2^ (%)	P(Q test)	Model	OR (95% CI)	Z score	P (Z)
**rs1043618**	**Mixed population**	5	615/925	G vs. C	19.7	0.29	F	1.150 (0.978, 1.351)	1.70	0.090
(G > C)				GG vs. CC	10.6	0.35	F	1.301 (0.909, 1.863)	1.44	0.151
				GG vs. GC	44.0	0.13	F	1.151 (0.912, 1.454)	1.18	0.236
				GG vs. GC + CC	39.5	0.16	F	1.188 (0.952, 1.484)	1.53	0.127
				GG + GC vs. CC	5.1	0.38	F	1.228 (0.879, 1.716)	1.21	0.228
	**Asian subgroup**	3	406/709	G vs. C	18.2	0.29	F	1.092 (0.897, 1.330)	0.88	0.381
				GG vs. CC	29.4	0.24	F	1.185(0.773, 1.815)	0.78	0.436
				GG vs. GC	54.5	0.11	F	1.064(0.799, 1.416)	0.42	0.671
				GG vs. GC + CC	42.6	0.17	F	1.099 (0.839, 1.440)	0.68	0.494
				GG + GC vs. CC	45.4	0.16	F	1.165 (0.784, 1.729)	0.76	0.450
	**Caucasian subgroup**	2	209/216	G vs. C	42.9	0.19	F	1.278 (0.964, 1.694)	1.70	0.088
				GG vs. CC	4.0	0.31	F	1.633(0.909, 1.863)	1.44	0.150
				GG vs. GC	44.7	0.18	F	1.349 (0.900, 2.023)	1.45	0.147
				GG vs. GC + CC	52.2	0.15	F	1.398(0.947, 2.064)	1.69 1.20	0.0910.228
				GG + GC vs. CC	0.0	0.54	F	1.403 (0.748, 2.633)	1.06	0.291
	**High quality**	4	525/828	G vs. C	0.0	0.48	F	1.089(0.914, 1.297)	0.96	0.339
	**subgroup**			GG vs. CC	0.0	0.42	F	1.191(0.812, 1.748)	0.89	0.371
				GG vs. GC	31.8	0.22	F	1.060(0.823, 1.366)	0.45	0.653
				GG vs. GC + CC	14.0	0.322	F	1.094(0.860, 1.391)	0.73	0.464
										
				GG + GC vs. CC	18.0	0.301	F	1.169(0.819, 1.669)	0.86	0.389
**rs2227956**	**Mixed population**	4	578/592	A vs. G	63.7	0.04	F	0.826(0.661, 1.033)	1.67	0.0940.213
(A > G)				AA vs. GG	62.4	0.05	F	0.549 (0.090, 3.348)	0.65	0.516
				AA vs. AG	27.1	0.25	F	0.802 (0.619, 1.040)	1.67	0.096
				AA vs. AG + GG	50.7	0.11	F	0.801(0.622, 1.031)	1.72	0.085
				AA + AG vs. GG	60.3	0.06	F	0.795(0.352, 1.795)	0.55	0.582
	**Asian subgroup**	2	369/377	A vs. G	0.0	0.86	F	1.064 (0.802, 1.410)	0.43	0.668
				AA vs. GG	41.3	0.19	F	2.328 (0.701, 7.727)	1.38	0.168
				AA vs. AG	0.0	0.33	F	0.959 (0.693, 1.327)	0.25	0.800
				AA vs. AG + GG	0.0	0.52	F	1.011 (0.734, 1.389)	0.07	0.945
				AA + AG vs. GG	47.7	0.17	F	2.335 (0.711, 7.671)	1.40	0.162
	**Caucasian subgroup**	2	209/215	**A vs. G**	0.0	0.90	F	**0.535 (0.368, 0.779)**	3.26	**0.001**
				**AA vs. GG**	0.0	0.58	F	**0.135 (0.024, 0.764)**	2.26	**0.024**
				**AA vs. AG**	0.0	0.99	F	**0.585 (0.379, 0.903)**	2.42	**0.016**
				**AA vs. AG + GG**	0.0	0.91	F	**0.531 (0.347, 0.812)**	2.92	**0.004**
				**AA + AG vs. GG**	0.0	0.60	F	**0.157 (0.028, 0.889)**	2.09	**0.036**
	**High quality**	3	470/494	A vs. G	55.9	0.10	F	0.921(0.718, 1.182)	0.65	0.514
	**subgroup**			AA vs. GG	59.4	0.09	F	1.215(0.476, 3.103)	0.41	0.684
				AA vs. AG	29.5	0.24	F	0.861(0.646, 1.147)	1.02	0.307
				AA vs. AG + GG	44.3	0.17	F	0.882(0.667, 1.166)	0.88	0.377
				AA + AG vs. GG	58.5	0.09	F	1.269(0.497, 3.237)	0.50	0.618
										
**rs1061581**	**Mixed population**	3	301/315	**A vs. G**	0.0	0.62	F	**1.322 (1.046, 1.671)**	2.34	**0.019**
(A > G)				**AA vs. GG**	0.0	0.50	F	**1.926 (1.104, 3.359)**	2.31	**0.021**
				AA vs. AG	43.7	0.17	F	1.378 (0.981,1.937)	1.85	0.065
				**AA vs. AG + GG**	27.7	0.25	F	**1.455 (1.048, 2.019)**	2.24	**0.025**
				AA + AG vs. GG	0.0	0.38	F	1.500 (0.901, 2.496)	1.56	0.119
	**Caucasian subgroup**	2	251/264	**A vs. G**	0.0	0.33	F	**1.342 (1.017, 1.771)**	2.08	**0.037**
	(HWE P > 0.05			AA vs. GG	0.0	0.49	F	1.679 (0.907, 3.109)	1.65	0.099
	subgroup)			**AA vs. AG**	41.9	0.19	F	**1.636 (1.073, 2.493)**	2.29	**0.022**
				**AA vs. AG + GG**	41.0	0.19	F	**1.648 (1.100, 2.468)**	2.42	**0.015**
				AA + AG vs. GG	0.0	0.87	F	1.262 (0.720, 2.210)	0.81	0.416
	**High quality**	2	107/217	A vs. G	0.0	0.81	F	1.222(0.920, 1.624)	1.38	0.167
	**subgroup**			AA vs. GG	21.7	0.26	F	1.814(0.912, 3.606)	1.70	0.090
				AA vs. AG	0.0	0.56	F	1.124(0.748, 1.689)	0.56	0.572
				AA vs. AG + GG	0.0	0.25	F	1.217(0.823, 1.800)	0.98	0.326
				AA + AG vs. GG	46.0	0.17	F	1.599(0.848, 3.014)	1.45	0.147
										
**rs2075800**	**Asian**	2	313/608	C vs. T	0.0	0.89	F	0.812 (0.649, 1.017)	1.82	0.069
(C > T)				CC vs. TT	0.0	0.84	F	0.612 (0.370, 1.013)	1.91	0.056
				CC vs. CT	0.0	0.69	F	0.872 (0.631, 1.206)	0.83	0.408
				CC vs. CT + TT	0.0	0.75	F	0.811 (0.596, 1.103)	1.33	0.182
				CC + CT vs. TT	0.0	0.73	F	0.658 (0.410, 1.055)	1.74	0.082
**rs2763979**	**Asian**	2	313/608	C vs. T	71.7	0.06	F	1.153(0.921, 1.444)	1.24	0.215
(C > T)				CC vs. TT	44.6	0.18	F	1.561 (0.950, 2.564)	1.76	0.079
				CC vs. CT	0.0	0.40	F	1.003 (0.724, 1.389)	0.02	0.988
				CC vs. CT + TT	50.0	0.16	F	1.085(0.795, 1.479)	0.51	0.608
				CC + CT vs. TT	34.4	0.22	F	1.550 (0.975, 2.464)	1.85	0.064

F: fixed-effects model. R: random-effects model. P < 0.05, showing statistically significant difference.

The authors provided the following addition to the discussion of the study’s limitations (Discussion, add to end of paragraph 4):

Limitation 4: All the effect sizes (ORs and 95%CIs) are pooled under fixed-effect model. However, a limitation of fixed-effect model is that the pooled effect size (ORs and 95%CIs) is only a best estimate of the effects of the included studies, which has no inference about a wider population. The reason why we did not perform a random effect analysis is that the number of included studies is small, which will take poor precision to the estimate of the variance between studies and leads to an incorrect application of the model. Limitation 5: Publication bias is difficult to estimate in this meta-analysis due to the small number of included studies. Because of some subjectivity when the funnel plot is used to judge publication bias, the funnel plots can only be for reference. Also because of the small number of studies included, the Egger’s test is severely limited in its ability to detect publication bias, so we cannot make any conclusions about the existence of publication bias based on the results of the Egger’s test.

The protocol for the meta-analysis was not registered but has been provided in S1 File. The results of the Q test for heterogeneity is in S6 File.

The statistical reviewer consulted in this case evaluated the reanalyses and reviewed the updates and clarifications outlined above and advised that the results were consistent with the results and conclusions reported in the article.

## Supporting information

S1 Fig The forest plots of subgroup analysis based on the ethnicity and the association between rs2227956 and NIHL susceptibility in different genetic models. (TIF)

S2 Table The MAF and LD pattern of the four investigated SNPs in HSP70 genes in different populations. (DOCX)

S1 File Study protocol. (DOCX)

S2 File Funnel plot results. Note that these results should be interpreted with caution in light of the small number of included studies.
(TIF)

S3 File Forest plots showing the meta-analysis results reporting in [1], including random and fixed effects analyses. (TIF)

S4 File Forest plots showing results of post-publication reanalyses using only fixed effects models. (TIF)

S5 File Data extracted on workplace, standard of noise exposure, diagnosis criteria of hearing impairment or NIHL susceptibility, and standard of control group. (DOCX)

S6 File Results of the Q test in the heterogeneity analysis. (DOCX)
