# Supplementary material for: Association of polymorphisms in heat shock protein 70 genes with the susceptibility to noise-induced hearing loss: A meta-analysis
Source: PLoS One. 2017 Nov 16;12(11):e0188195. doi: 10.1371/journal.pone.0188195 (PMC5689837; doi:10.1371/journal.pone.0188195)
Supplement: S1 Diagram — (DOC) [file pone.0188195.s001.doc]

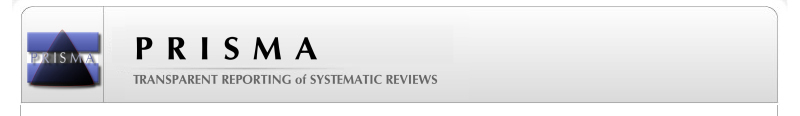
**PRISMA 2009 Flow Diagram**

**Screening**

**Included**

**Eligibility**

**Identification**

Records identified through database searching
(n = 143)

Additional records identified through other sources
(n = 0)

Records after duplicates removed
(n = 54)

Records screened
(n = 54)

Records excluded (n = 21): not about the relationship between HSP70 and NIHL

Full-text articles assessed for eligibility
(n = 33)

Full-text articles excluded, with reasons (n = 29):

- not about the SNPs in HSP70 genes (n = 15),
- review or not case-control study (n = 14).

Studies included in qualitative synthesis
(n = 4)

Studies included in quantitative synthesis (meta-analysis)
(n = 4)
