# Supplementary material for: Association of polymorphisms in heat shock protein 70 genes with the susceptibility to noise-induced hearing loss: A meta-analysis
Source: PLoS One. 2017 Nov 16;12(11):e0188195. doi: 10.1371/journal.pone.0188195 (PMC5689837; doi:10.1371/journal.pone.0188195)
Supplement: S1 Appendix — (DOCX) [file pone.0188195.s002.docx]

**Appendix S2 List of excluded studies with reasons.**

Article [1] was a review and excluded due to its nature.

Article [2] was not about the relationship between HSP70 and NIHL.

Article [3] was not about the relationship between HSP70 and NIHL.

Article [4] was not about the relationship between HSP70 and NIHL.

Article [5] was not about the relationship between HSP70 and NIHL.

Article [6] was not about the relationship between HSP70 and sudden hearing loss.

Article [7] was a review and excluded due to its nature.

Article [8] focus on the variation of hsp70 in cisplatin-induced hearing loss.

Article [9] was a review and excluded due to its nature.

Article [10] focus on the relationship between HSP70 and systemic lupus erythematosus but NIHL.

Article [11] studied HSP70 gene expression pattern rather than genetic polymorphisms in NIHL.

Article [12] studied HSP70 gene expression pattern rather than genetic polymorphisms in NIHL.

Article [13] did not investigate the HSP70 polymorphisms in NIHL.

Article [14] did not focus on the relationship between HSP70 and NIHL.

Article [15] did not focus on the relationship between HSP70 and NIHL.

Article [16] was a review and excluded due to its nature.

Article [17] did not focus on the relationship between HSP70 and NIHL.

Article [18] did not focus on the polymorphisms of HSP70 gene in NIHL.

Article [19] was not about the relationship between HSP70 and NIHL.

Article [20] did not investigate the HSP70 polymorphisms in NIHL.

Article [21] was not about the relationship between HSP70 and NIHL.

Article [22] did not focus on the polymorphisms of HSP70 gene in NIHL.

Article [23] did not investigate the HSP70 polymorphisms in NIHL.

Article [24] did not investigate the HSP70 polymorphisms in NIHL.

Article [25] studied HSP70 gene expression pattern rather than genetic polymorphisms in NIHL.

Article [26] did not focus on the polymorphisms of HSP70 gene in NIHL.

Article [27] focus on the polymorphisms associated with K+ Ion Circulation in the Inner Ear rather than HSP70 in NIHL subjects.

Article [28] focus on the variation of HSP70 expression but the polymorphisms in HSP70 gene.

Article [29] was a review and excluded due to its nature.

Article [30] was a review and excluded due to its nature.

Article [31] focus on the variation of HSP70 expression in a endotoxin-induced cochlea impairment model.

Article [32] was not a case-control study and excluded due to its nature.

Article [33] did not focus on the polymorphisms of HSP70 gene in NIHL.

Article [34] studied HSP70 gene expression pattern rather than genetic polymorphisms in NIHL.

Article [35] focus on the function of HSP70 in gentamicin-induced vestibular hair cell death.

Article [36] studied the function of HSP70 in aminoglycoside-induced hearing loss.

Article [37] was not a case-control study and excluded due to its nature.

Article [38] was not about the relationship between HSP70 and NIHL.

Article [39] did not focus on the HSP70 polymorphisms in NIHL.

Article [40] was a review and excluded due to its nature.

Article [41] was not about the relationship between HSP70 and NIHL.

Article [42] focus on the variation of HSP70 antibody but HSP70 polymorphisms in NIHL.

Article [43] focus on the variation of HSP70 antibody but HSP70 polymorphisms in NIHL.

Article [44] focus on the variation of hsp70 protein expression rather than HSP70 polymorphisms in NIHL.

Article [45] did not focus on the HSP70 polymorphisms in NIHL.

Paper [46] was an abstract and data are insufficient for analysis.

Paper [47] was an abstract and data are insufficient for analysis.

Paper [48] was an abstract and data are insufficient for analysis.

Paper [49] was an abstract and data are insufficient for analysis.

Paper [50] was an abstract of a poster and data are insufficient for analysis.

1. Altschuler RA, Fairfield D, Cho Y, Leonova E, Benjamin IJ, Miller JM, et al. Stress pathways in the rat cochlea and potential for protection from acquired deafness. Audiology & neuro-otology. 2002;7(3):152-6. doi: 58301. PubMed PMID: 12053136.

2. Asoh S, Ohta S. PTD-mediated delivery of anti-cell death proteins/peptides and therapeutic enzymes. Adv Drug Deliv Rev. 2008;60(4-5):499-516. doi: 10.1016/j.addr.2007.09.011. PubMed PMID: 18093693.

3. Barden EK, Rellinger EA, Ortmann AJ, Ohlemiller KK. Inheritance Patterns of Noise Vulnerability and "Protectability" in (C57BL/6J x CBA/J) Fl Hybrid Mice. Journal of the American Academy of Audiology. 2012;23(5):332-40.

4. Canlon B, Meltser I, Johansson P, Tahera Y. Glucocorticoid receptors modulate auditory sensitivity to acoustic trauma. Hearing research. 2007;226(1-2):61-9. doi: 10.1016/j.heares.2006.05.009. PubMed PMID: 16843624.

5. Charitidi K, Meltser I, Tahera Y, Canlon B. Functional responses of estrogen receptors in the male and female auditory system. Hearing research. 2009;252(1-2):71-8. doi: 10.1016/j.heares.2008.12.009. PubMed PMID: 19450435.

6. Chien CY, Chang NC, Tai SY, Wang LF, Wu MT, Ho KY. Heat shock protein 70 gene polymorphisms in sudden sensorineural hearing loss. Audiology & neuro-otology. 2012;17(6):381-5. doi: 10.1159/000341815. PubMed PMID: 22922572.

7. Clifford RE, Hoffer M, Rogers R. The Genomic Basis of Noise-induced Hearing Loss: A Literature Review Organized by Cellular Pathways. Otology & neurotology : official publication of the American Otological Society, American Neurotology Society [and] European Academy of Otology and Neurotology. 2016;37(8):e309-16. doi: 10.1097/MAO.0000000000001073. PubMed PMID: 27518140.

8. Coling DE, Ding D, Young R, Lis M, Stofko E, Blumenthal KM, et al. Proteomic analysis of cisplatin-induced cochlear damage: methods and early changes in protein expression. Hearing research. 2007;226(1-2):140-56. doi: 10.1016/j.heares.2006.12.017. PubMed PMID: 17321087.

9. Fetoni AR, Picciotti PM, Paludetti G, Troiani D. Pathogenesis of presbycusis in animal models: a review. Exp Gerontol. 2011;46(6):413-25. doi: 10.1016/j.exger.2010.12.003. PubMed PMID: 21211561.

10. Furnrohr BG, Wach S, Kelly JA, Haslbeck M, Weber CK, Stach CM, et al. Polymorphisms in the Hsp70 gene locus are genetically associated with systemic lupus erythematosus. Ann Rheum Dis. 2010;69(11):1983-9. doi: 10.1136/ard.2009.122630. PubMed PMID: 20498198; PubMed Central PMCID: PMC3002760.

11. Gong TW, Fairfield DA, Fullarton L, Dolan DF, Altschuler RA, Kohrman DC, et al. Induction of heat shock proteins by hyperthermia and noise overstimulation in hsf1 -/- mice. Journal of the Association for Research in Otolaryngology : JARO. 2012;13(1):29-37. doi: 10.1007/s10162-011-0289-9. PubMed PMID: 21932106; PubMed Central PMCID: PMC3254713.

12. Gratton MA, Eleftheriadou A, Garcia J, Verduzco E, Martin GK, Lonsbury-Martin BL, et al. Noise-induced changes in gene expression in the cochleae of mice differing in their susceptibility to noise damage. Hearing research. 2011;277(1-2):211-26. doi: 10.1016/j.heares.2010.12.014. PubMed PMID: 21187137; PubMed Central PMCID: PMC3098916.

13. Grondin Y, Bortoni ME, Sepulveda R, Ghelfi E, Bartos A, Cotanche D, et al. Genetic Polymorphisms Associated with Hearing Threshold Shift in Subjects during First Encounter with Occupational Impulse Noise. PloS one. 2015;10(6):e0130827. Epub 2015/06/30. doi: 10.1371/journal.pone.0130827. PubMed PMID: 26121033; PubMed Central PMCID: PMC4488244.

14. Gross M, Eliashar R, Ben-Yaakov A, Ulmansky R, Elidan J. Prevalence and clinical significance of anticardiolipin, anti-beta(2)-glycoprotein-1, and anti-heat shock protein-70 autoantibodies in sudden sensorineural hearing loss. Audiology and Neuro-Otology. 2008;13(4):231-8. doi: 10.1159/000115432. WOS:000255895800003.

15. Kim YH, Song JJ, Kim YC, Park KT, Lee JH, Choi JM, et al. Geranylgeranylacetone ameliorates acute cochlear damage caused by 3-nitropropionic acid. Neurotoxicology. 2010;31(3):317-25. doi: 10.1016/j.neuro.2010.03.001. PubMed PMID: 20226206.

16. Konings A, Van Laer L, Van Camp G. Genetic studies on noise-induced hearing loss: a review. Ear Hear. 2009;30(2):151-9. doi: 10.1097/AUD.0b013e3181987080. PubMed PMID: 19194285.

17. Kowalski TJ, Pawelczyk M, Rajkowska E, Dudarewicz A, Sliwinska-Kowalska M. Genetic variants of CDH23 associated with noise-induced hearing loss. Otology & neurotology : official publication of the American Otological Society, American Neurotology Society [and] European Academy of Otology and Neurotology. 2014;35(2):358-65. doi: 10.1097/MAO.0b013e3182a00332. PubMed PMID: 24448297.

18. Maeda Y, Fukushima K, Kariya S, Orita Y, Nishizaki K. Dexamethasone Regulates Cochlear Expression of Deafness-associated Proteins Myelin Protein Zero and Heat Shock Protein 70, as Revealed by iTRAQ Proteomics. Otology & neurotology : official publication of the American Otological Society, American Neurotology Society [and] European Academy of Otology and Neurotology. 2015;36(7):1255-65. Epub 2015/04/02. doi: 10.1097/MAO.0000000000000748. PubMed PMID: 25830874.

19. Mastorci F, Vicentini M, Viltart O, Manghi M, Graiani G, Quaini F, et al. Long-term effects of prenatal stress: changes in adult cardiovascular regulation and sensitivity to stress. Neurosci Biobehav Rev. 2009;33(2):191-203. doi: 10.1016/j.neubiorev.2008.08.001. PubMed PMID: 18771688.

20. May LA, Kramarenko, II, Brandon CS, Voelkel-Johnson C, Roy S, Truong K, et al. Inner ear supporting cells protect hair cells by secreting HSP70. J Clin Invest. 2013;123(8):3577-87. doi: 10.1172/JCI68480. PubMed PMID: 23863716; PubMed Central PMCID: PMC3967657.

21. Mijovic T, Zeitouni A, Colmegna I. Autoimmune sensorineural hearing loss: the otology-rheumatology interface. Rheumatology (Oxford). 2013;52(5):780-9. doi: 10.1093/rheumatology/ket009. PubMed PMID: 23436581.

22. Mikuriya T, Sugahara K, Takemoto T, Tanaka K, Takeno K, Shimogori H, et al. Geranylgeranylacetone, a heat shock protein inducer, prevents acoustic injury in the guinea pig. Brain research. 2005;1065(1-2):107-14. Epub 2005/11/29. doi: 10.1016/j.brainres.2005.10.045. PubMed PMID: 16309629.

23. Momin SR, Melki SJ, Obokhare JO, Fares SA, Semaan MT, Megerian CA. Hearing preservation in Guinea pigs with long-standing endolymphatic hydrops. Otology & neurotology : official publication of the American Otological Society, American Neurotology Society [and] European Academy of Otology and Neurotology. 2011;32(9):1583-9. doi: 10.1097/MAO.0b013e3182382a64. PubMed PMID: 22015942; PubMed Central PMCID: PMC3220888.

24. Monzack EL, Cunningham LL. Lead roles for supporting actors: critical functions of inner ear supporting cells. Hearing research. 2013;303(C):20-9. doi: 10.1016/j.heares.2013.01.008. PubMed PMID: 23347917; PubMed Central PMCID: PMC3648608.

25. Ohlemiller KK. Recent findings and emerging questions in cochlear noise injury. Hearing research. 2008;245(1-2):5-17. doi: 10.1016/j.heares.2008.08.007. PubMed PMID: 18790034; PubMed Central PMCID: PMC2610263.

26. Ohlemiller KK, Rybak Rice ME, Rosen AD, Montgomery SC, Gagnon PM. Protection by low-dose kanamycin against noise-induced hearing loss in mice: dependence on dosing regimen and genetic background. Hearing research. 2011;280(1-2):141-7. doi: 10.1016/j.heares.2011.05.007. PubMed PMID: 21645602; PubMed Central PMCID: PMC3175505.

27. Pawelczyk M, Van Laer L, Fransen E, Rajkowska E, Konings A, Carlsson PI, et al. Analysis of gene polymorphisms associated with K ion circulation in the inner ear of patients susceptible and resistant to noise-induced hearing loss. Ann Hum Genet. 2009;73(Pt 4):411-21. doi: 10.1111/j.1469-1809.2009.00521.x. PubMed PMID: 19523148.

28. Roy S, Ryals MM, Van den Bruele AB, Fitzgerald TS, Cunningham LL. Sound preconditioning therapy inhibits ototoxic hearing loss in mice. J Clin Invest. 2013;123(11):4945-9. doi: 10.1172/JCI71353. PubMed PMID: 24216513; PubMed Central PMCID: PMC3809804.

29. Shi X. Pathophysiology of the cochlear intrastrial fluid-blood barrier (review). Hearing research. 2016;338:52-63. doi: 10.1016/j.heares.2016.01.010. PubMed PMID: 26802581; PubMed Central PMCID: PMC5322264.

30. Sliwinska-Kowalska M, Pawelczyk M. Contribution of genetic factors to noise-induced hearing loss: a human studies review. Mutation research. 2013;752(1):61-5. Epub 2012/12/05. doi: 10.1016/j.mrrev.2012.11.001. PubMed PMID: 23207014.

31. Sone M, Hayashi H, Yamamoto H, Hoshino T, Mizushima T, Nakashima T. Upregulation of HSP by geranylgeranylacetone protects the cochlear lateral wall from endotoxin-induced inflammation. Hearing research. 2005;204(1-2):140-6. Epub 2005/06/01. doi: 10.1016/j.heares.2005.01.012. PubMed PMID: 15925199.

32. Steyger PP. Translating In Vitro Data into Auditory Protection. Hearing Journal. 2014;67(4):20,2.

33. Sugahara K, Inouye S, Izu H, Katoh Y, Katsuki K, Takemoto T, et al. Heat shock transcription factor HSF1 is required for survival of sensory hair cells against acoustic overexposure. Hearing research. 2003;182(1-2):88-96. Epub 2003/09/02. PubMed PMID: 12948605.

34. Sun W, Zhang L, Lu J, Yang G, Laundrie E, Salvi R. Noise exposure-induced enhancement of auditory cortex response and changes in gene expression. Neuroscience. 2008;156(2):374-80. Epub 2008/08/21. doi: 10.1016/j.neuroscience.2008.07.040. PubMed PMID: 18713646; PubMed Central PMCID: PMC2573047.

35. Takumida M, Anniko M. Heat shock protein 70 delays gentamicin-induced vestibular hair cell death. Acta Otolaryngol. 2005;125(1):23-8. PubMed PMID: 15799569.

36. Taleb M, Brandon CS, Lee FS, Harris KC, Dillmann WH, Cunningham LL. Hsp70 inhibits aminoglycoside-induced hearing loss and cochlear hair cell death. Cell stress & chaperones. 2009;14(4):427-37. doi: 10.1007/s12192-008-0097-2. PubMed PMID: 19145477; PubMed Central PMCID: PMC2728278.

37. Themann CMA, Suter AHPD, Stephenson MRPD. National Research Agenda for the Prevention of Occupational Hearing Loss-Part 1. Seminars in Hearing. 2013;34(3):145-207.

38. Vazzana M, Celi M, Arizza V, Calandra G, Buscaino G, Ferrantelli V, et al. Noise elicits hematological stress parameters in Mediterranean damselfish (Chromis chromis, perciformes): A mesocosm study. Fish Shellfish Immunol. 2017;62:147-52. doi: 10.1016/j.fsi.2017.01.022. PubMed PMID: 28108343.

39. Wang B, Ding E, Shen H, Wang J, Sun K, Chen S, et al. Association of TagSNP in lncRNA HOTAIR with susceptibility to noise-induced hearing loss in a Chinese population. Hearing research. 2017;347:41-6. doi: 10.1016/j.heares.2017.02.007. PubMed PMID: 28223190.

40. Warchol ME. Cellular mechanisms of aminoglycoside ototoxicity. Curr Opin Otolaryngol Head Neck Surg. 2010;18(5):454-8. doi: 10.1097/MOO.0b013e32833e05ec. PubMed PMID: 20717031.

41. Yamahara K, Yamamoto N, Nakagawa T, Ito J. Insulin-like growth factor 1: A novel treatment for the protection or regeneration of cochlear hair cells. Hearing research. 2015;330(Pt A):2-9. doi: 10.1016/j.heares.2015.04.009. PubMed PMID: 25937136.

42. Yang M, Zheng J, Yang Q, Yao H, Chen Y, Tan H, et al. Frequency-specific association of antibodies against heat shock proteins 60 and 70 with noise-induced hearing loss in Chinese workers. Cell stress & chaperones. 2004;9(2):207-13. doi: 10.1379/csc-12r.1. PubMed PMID: 15497506; PubMed Central PMCID: PMC1065299.

43. Yuan J, Yang M, Yao H, Zheng J, Yang Q, Chen S, et al. Plasma antibodies to heat shock protein 60 and heat shock protein 70 are associated with increased risk of electrocardiograph abnormalities in automobile workers exposed to noise. Cell stress & chaperones. 2005;10(2):126-35. Epub 2005/07/26. PubMed PMID: 16038409; PubMed Central PMCID: PMC1176471.

44. Zuo H, Cui B, She X, Wu M. Changes in Guinea pig cochlear hair cells after sound conditioning and noise exposure. Journal of occupational health. 2008;50(5):373-9. Epub 2008/07/26. PubMed PMID: 18654041.

45. Zuo HY, Wu MQ, Cui B, She XJ. [The mechanism of protection by sound conditioning from acoustic trauma]. Chinese journal of applied physiology. 2005;21(4):462-5. PubMed PMID: 21180176.

46. Current World Literature. Current Opinion in Anaesthesiology. 2000;13(2):219-50.

47. [Abstracts of the 40th Easter/Western Regional Meeting, Japanese Society of Nephrology. 2010. Japan]. Nihon Jinzo Gakkai Shi. 2010;52(6):651-868. PubMed PMID: 20928953.

48. Abst D-FreeCommPosters. Medicine & Science in Sports & Exercise. 2012;44(5S) Supplement(2):267-958.

49. Abstracts and Highlight Papers of the 31st Annual European Society of Regional Anaesthesia (ESRA) Congress 2012. Regional Anesthesia & Pain Medicine September/October. 2012;37(5):E1-E311.

50. Poster Session. Movement Disorders. 2016;31 Abstracts of the Twentieth International Congress of Parkinson's Disease and Movement(DisordersS2):S1-S697.
