# Supplementary material for: Association of polymorphisms in heat shock protein 70 genes with the susceptibility to noise-induced hearing loss: A meta-analysis
Source: PLoS One. 2017 Nov 16;12(11):e0188195. doi: 10.1371/journal.pone.0188195 (PMC5689837; doi:10.1371/journal.pone.0188195)
Supplement: S1 Table — (DOCX) [file pone.0188195.s003.docx]

| S1 Table. Publication bias analysis (Egger’s test) | | | | |
| --- | --- | --- | --- | --- |
| SNP | **Genetic model** | **Egger’s test** | | |
|  |  | **p** | **95% CI** | **Publication bias** |
| rs1043618 | G vs. C | 0.880 | -6.128, 6.797 | NO |
| (G > C) | GG vs. CC | 0.625 | -5.084, 3.602 | NO |
|  | GG vs. GC | 0.229 | -2.982, 8.341 | NO |
|  | GG vs. GC + CC | 0.411 | -4.230, 7.848 | NO |
|  | GG + GC vs. CC | 0.284 | -4.964, 2.081 | NO |
| rs2227956 | A vs. G | 0.325 | -20.297, 10.908 | NO |
| (A > G) | AA vs. GG | 0.297 | -43.555, 31.795 | NO |
|  | AA vs. AG | 0.702 | -15.415, 12.543 | NO |
|  | AA vs. AG + GG | 0.497 | -18.003, 12.223 | NO |
|  | AA + AG vs. GG | 0.263 | -39.319, 27.348 | NO |
| rs1061581 | A vs. G | 0.707 | -93.618, 101.234 | NO |
| (A > G) | AA vs. GG | 0.206 | -10.465, 16.868 | NO |
|  | AA vs. AG | 0.331 | -130.308, 171.773 | NO |
|  | AA vs. AG + GG | 0.325 | -107.886, 143.187 | NO |
|  | AA + AG vs. GG | 0.097 | -3.325, 10.322 | NO |
| When p < 0.05 or 95% CI does not contain 0, publication bias was considered existed. NA: not available. NO: no publication bias. | | | | |
