# Supplementary material for: Association of polymorphisms in heat shock protein 70 genes with the susceptibility to noise-induced hearing loss: A meta-analysis
Source: PLoS One. 2017 Nov 16;12(11):e0188195. doi: 10.1371/journal.pone.0188195 (PMC5689837; doi:10.1371/journal.pone.0188195)
Supplement: S2 Table — (DOCX) [file pone.0188195.s004.docx]

**Supplementary Table 2.**The minor allele frequency (MAF) and [linkage](file:///F:\%E6%9C%89%E9%81%93%E8%AF%8D%E5%85%B8\Dict\7.0.1.0227\resultui\dict\?keyword=linkage) [disequilibrium](file:///F:\%E6%9C%89%E9%81%93%E8%AF%8D%E5%85%B8\Dict\7.0.1.0227\resultui\dict\?keyword=disequilibrium) (LD) pattern of the four investigated SNPs in HSP70 genes from different populations (Shown were *r^2^ and D’* values from 1000 Genome project)

| **The MAF the four investigated SNPs in HSP70 genes from different populations** | | | | |
| --- | --- | --- | --- | --- |
| MAF | rs1043618(C) | rs2763979(T) | rs2075800(T) | rs2227956(A) |
| CHB | 0.301 | 0.3495 | 0.3592 | 0.8204 |
| CHS | 0.1952 | 0.1476 | 0.4810 | 0.7667 |
| CEU | 0.3384 | 0.3081 | 0.3131 | 0.7576 |
| FIN | 0.4747 | 0.4444 | 0.3131 | 0.8434 |
| GBR | 0.3462 | 0.3132 | 0.3132 | 0.7912 |
| IBS | 0.3645 | 0.3598 | 0.4346 | 0.9252 |
| TSI | 0.4346 | 0.3925 | 0.3598 | 0.8832 |

The MAF of rs1061581 is not available. CHB: Han Chinese in Beijing, China; CHS: Southern Han Chinese; CEU: Utah residents with Northern and Western European ancestry; FIN: Finish in Finland; GBR: British in England and Scotland; IBS: Iberian Population in Spain; TSI: Toscani in Italia.

|  | **The MAF the four investigated SNPs in HSP70 genes from different populations** | | | | | | | | |
| --- | --- | --- | --- | --- | --- | --- | --- | --- | --- |
| Population | SNP | rs1043618 | | rs2763979 | | rs2075800 | | rs2227956 | |
|  |  | r^2^ | D' | r^2^ | D' | r^2^ | D' | r^2^ | D' |
| CHB | rs1043618 |  |  | 0.196731 | 0.495491 | 0.241352 | 0.999958 | 0.09424 | 0.999877 |
|  | rs2763979 |  |  |  |  | 0.301208 | 0.999978 | 0.082503 | 0.837458 |
|  | rs2075800 |  |  |  |  |  |  | 0.122718 | 0.999926 |
|  | rs2227956 |  |  |  |  |  |  |  |  |
| CHS | rs1043618 |  |  | 0.064236 | 0.299973 | 0.224792 | 0.999988 | 0.073734 | 0.999308 |
|  | rs2763979 |  |  |  |  | 0.131753 | 0.906106 | 0.052671 | 0.999649 |
|  | rs2075800 |  |  |  |  |  |  | 0.282003 | 0.999986 |
|  | rs2227956 |  |  |  |  |  |  |  |  |
| CEU | rs1043618 |  |  | 0.698259 | 0.895582 | 0.233158 | 0.999993 | 0.163639 | 0.999922 |
|  | rs2763979 |  |  |  |  | 0.202971 | 0.999969 | 0.142461 | 0.999927 |
|  | rs2075800 |  |  |  |  |  |  | 0.145854 | 0.999769 |
|  | rs2227956 |  |  |  |  |  |  |  |  |
| FIN | rs1043618 |  |  | 0.730645 | 0.908564 | 0.412038 | 0.999989 | 0.167768 | 0.999965 |
|  | rs2763979 |  |  |  |  | 0.364691 | 0.999979 | 0.148479 | 0.999920 |
|  | rs2075800 |  |  |  |  |  |  | 0.084617 | 0.999954 |
|  | rs2227956 |  |  |  |  |  |  |  |  |
| GBR | rs1043618 |  |  | 0.588588 | 0.826647 | 0.241395 | 0.999965 | 0.139675 | 0.999889 |
|  | rs2763979 |  |  |  |  | 0.171231 | 0.907457 | 0.120318 | 0.999935 |
|  | rs2075800 |  |  |  |  |  |  | 0.120330 | 0.999984 |
|  | rs2227956 |  |  |  |  |  |  |  |  |
| (continued) | | | | | | | | | |

| **The MAF the four investigated SNPs in HSP70 genes from different populations** (continued) | | | | | | | | | |
| --- | --- | --- | --- | --- | --- | --- | --- | --- | --- |
| Population | SNP | rs1043618 | | rs2763979 | | rs2075800 | | rs2227956 | |
|  |  | r^2^ | D' | r^2^ | D' | r^2^ | D' | r^2^ | D' |
| IBS | rs1043618 |  |  | 0.821878 | 0.915791 | 0.440774 | 0.999957 | NA | NA |
|  | rs2763979 |  |  |  |  | 0.431971 | 0.999985 | NA | NA |
|  | rs2075800 |  |  |  |  |  |  | 0.062102 | 0.999944 |
|  | rs2227956 |  |  |  |  |  |  |  |  |
| TSI | rs1043618 |  |  | 0.731648 | 0.932893 | 0.431962 | 0.999974 | 0.101661 | 0.999976 |
|  | rs2763979 |  |  |  |  | 0.363147 | 0.999973 | 0.085464 | 0.999966 |
|  | rs2075800 |  |  |  |  |  |  | 0.074338 | 0.999954 |
|  | rs2227956 |  |  |  |  |  |  |  |  |
| The LD pattern of rs1061581 is not available due to its unavailable MAF. CHB: Han Chinese in Beijing, China; CHS: Southern Han Chinese; CEU: Utah residents with Northern and Western European ancestry; FIN: Finish in Finland; GBR: British in England and Scotland; IBS: Iberian Population in Spain; TSI: Toscani in Italia. NA: not available. | | | | | | | | | |
