# Supplementary figures and images for: Association of polymorphisms in heat shock protein 70 genes with the susceptibility to noise-induced hearing loss: A meta-analysis
Source: PLoS One. 2017 Nov 16;12(11):e0188195. doi: 10.1371/journal.pone.0188195 (PMC5689837; doi:10.1371/journal.pone.0188195)

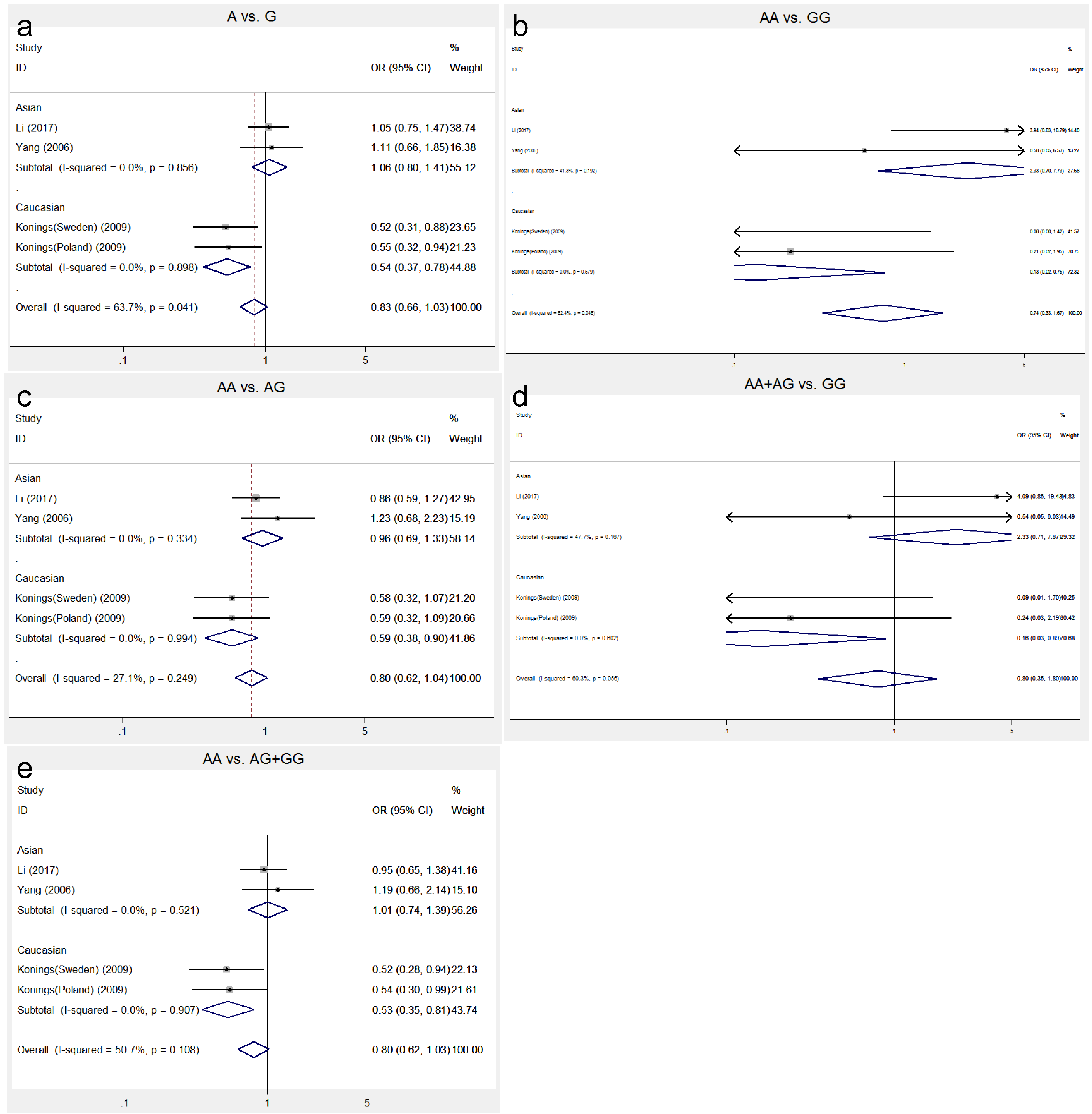

Supplement: S1 Fig — (TIF) [file pone.0188195.s007.tif]

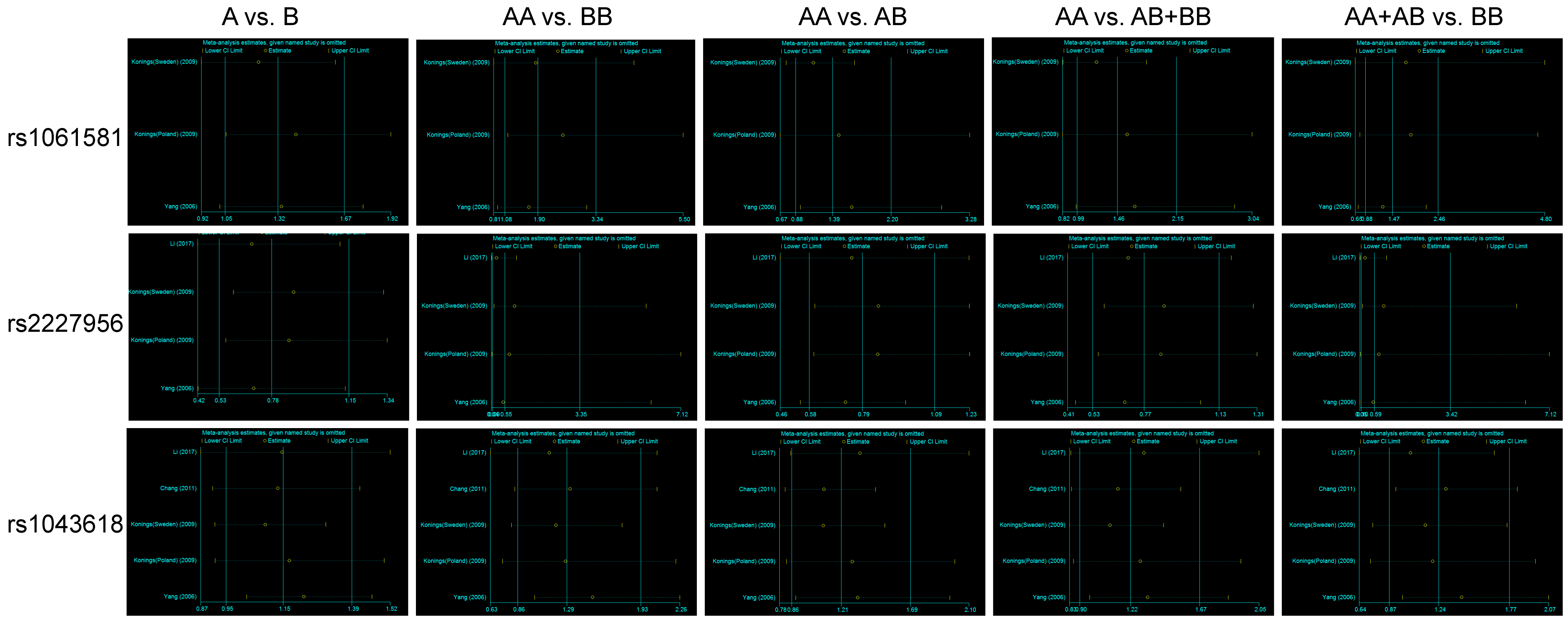

Supplement: S2 Fig — (TIF) [file pone.0188195.s008.tif]
