# Supplementary figures and images for: Correction: Association of polymorphisms in heat shock protein 70 genes with the susceptibility to noise-induced hearing loss: A meta-analysis
Source: PLoS One. 2020 Nov 17;15(11):e0242647. doi: 10.1371/journal.pone.0242647 (PMC7671542; doi:10.1371/journal.pone.0242647)

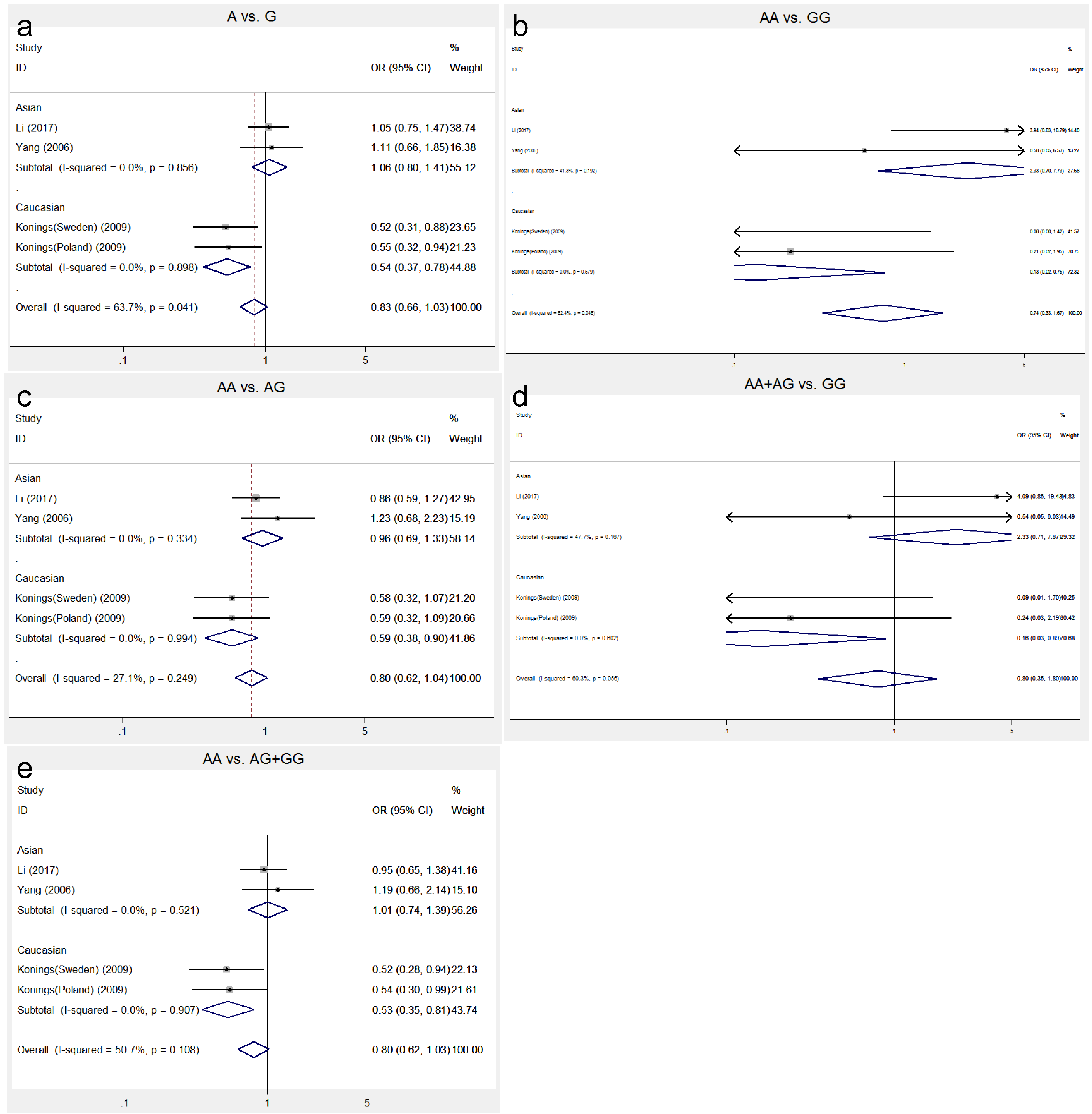

Supplement: S1 Fig — (TIF) [file pone.0242647.s001.tif]

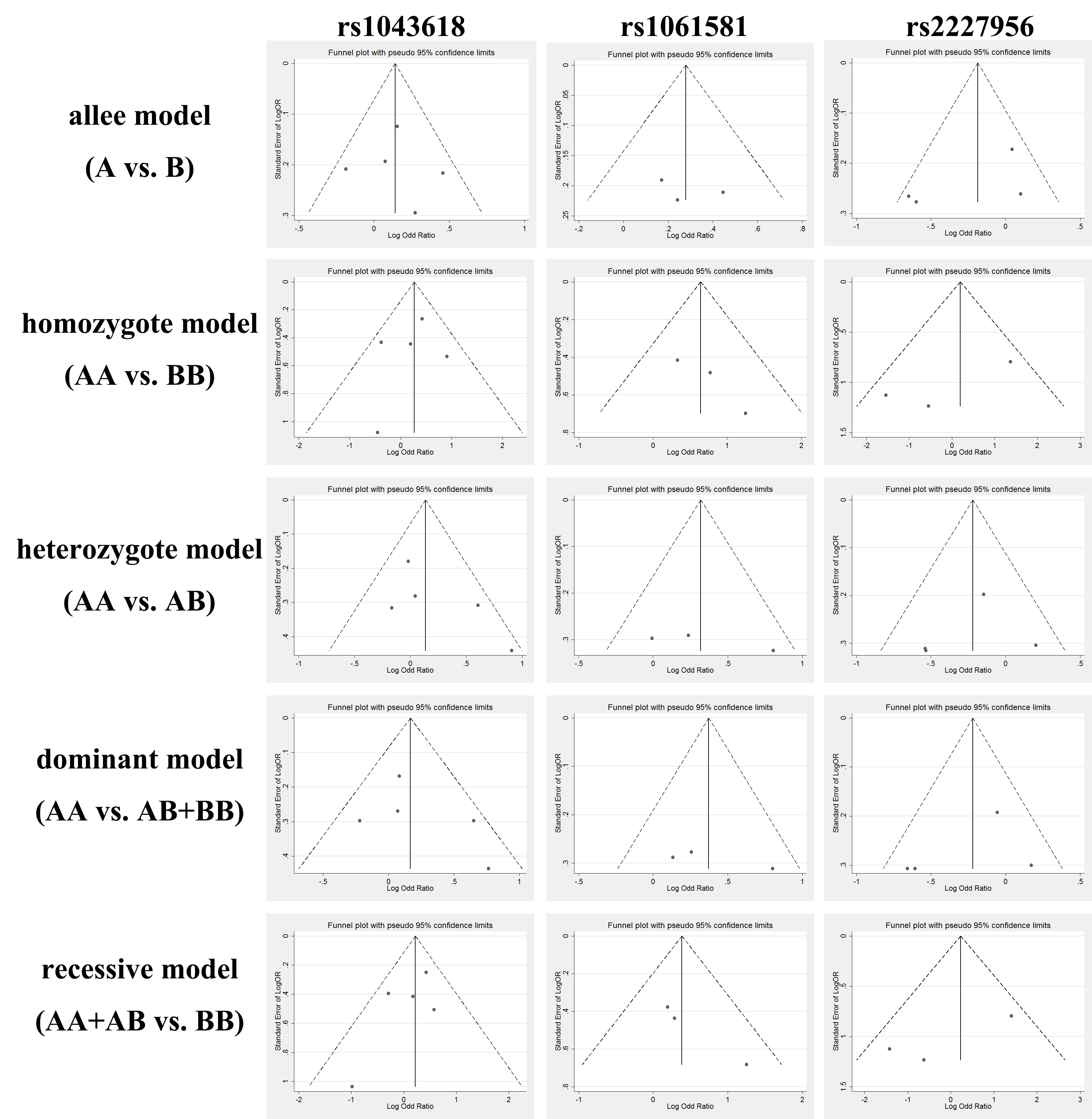

Supplement: S2 File — Note that these results should be interpreted with caution in light of the small number of included studies. (TIF) [file pone.0242647.s004.tif]
