# Supplementary material for: Correction: Association of polymorphisms in heat shock protein 70 genes with the susceptibility to noise-induced hearing loss: A meta-analysis
Source: PLoS One. 2020 Nov 17;15(11):e0242647. doi: 10.1371/journal.pone.0242647 (PMC7671542; doi:10.1371/journal.pone.0242647)
Supplement: S2 Table — (DOCX) [file pone.0242647.s002.docx]

**S2 Table.**The minor allele frequency (MAF) and [linkage](file:///F:\%E6%9C%89%E9%81%93%E8%AF%8D%E5%85%B8\Dict\7.0.1.0227\resultui\dict\?keyword=linkage) [disequilibrium](file:///F:\%E6%9C%89%E9%81%93%E8%AF%8D%E5%85%B8\Dict\7.0.1.0227\resultui\dict\?keyword=disequilibrium) (LD) pattern of the four investigated SNPs in *HSP70* genes from different populations (Shown were *r^2^ and D’* values from 1000 Genome project)

| **The MAF the four investigated SNPs in *HSP70* genes from different populations** | | | | |
| --- | --- | --- | --- | --- |
| MAF | rs1043618(C) | rs2763979(T) | rs2075800(T) | rs2227956(A) |
| CHB(Beijing) | 0.301 | 0.3495 | 0.3592 | 0.8204 |
| CHS(southern) | 0.1952 | 0.1476 | 0.481 | 0.7667 |
| CEU | 0.3384 | 0.3081 | 0.3131 | 0.7576 |

The MAF of rs1061581 is not available.

| **The LD pattern of the four investigated SNPs in *HSP70* genes from different populations** | | | | | | | | |
| --- | --- | --- | --- | --- | --- | --- | --- | --- |
| Han Chinese in Bejing, China (CHB) | | | | | | | | |
| CHB | rs1043618 | | rs2763979 | | rs2075800 | | rs2227956 | |
|  | r^2^ | D' | r^2^ | D' | r^2^ | D' | r^2^ | D' |
| rs1043618 |  |  | 0.196731 | 0.495491 | 0.241352 | 0.999958 | 0.09424 | 0.999877 |
| rs2763979 |  |  |  |  | 0.301208 | 0.999978 | 0.082503 | 0.837458 |
| rs2075800 |  |  |  |  |  |  | 0.122718 | 0.999926 |
| rs2227956 |  |  |  |  |  |  |  |  |
|  |  |  |  |  |  |  |  |  |
| Southern Han Chinese, China (CSH) | | | | | | | | |
| CHB | rs1043618 | | rs2763979 | | rs2075800 | | rs2227956 | |
|  | r^2^ | D' | r^2^ | D' | r^2^ | D' | r^2^ | D' |
| rs1043618 |  |  | 0.064236 | 0.299973 | 0.224792 | 0.999988 | 0.073734 | 0.999308 |
| rs2763979 |  |  |  |  | 0.131753 | 0.906106 | 0.052671 | 0.999649 |
| rs2075800 |  |  |  |  |  |  | 0.282003 | 0.999986 |
| rs2227956 |  |  |  |  |  |  |  |  |
|  |  |  |  |  |  |  |  |  |
| Utah residents with Northern and Western European ancestry (CEU) | | | | | | | | |
| CEU | rs1043618 | | rs2763979 | | rs2075800 | | rs2227956 | |
|  | r^2^ | D' | r^2^ | D' | r^2^ | D' | r^2^ | D' |
| rs1043618 |  |  | 0.698259 | 0.895582 | 0.233158 | 0.999993 | 0.163639 | 0.999922 |
| rs2763979 |  |  |  |  | 0.202971 | 0.999969 | 0.142461 | 0.999927 |
| rs2075800 |  |  |  |  |  |  | 0.145854 | 0.999769 |
| rs2227956 |  |  |  |  |  |  |  |  |
| The LD pattern of rs1061581 is not available due to its unavailable MAF. | | | | | | | | |
