# Supplementary material for: Correction: Association of polymorphisms in heat shock protein 70 genes with the susceptibility to noise-induced hearing loss: A meta-analysis
Source: PLoS One. 2020 Nov 17;15(11):e0242647. doi: 10.1371/journal.pone.0242647 (PMC7671542; doi:10.1371/journal.pone.0242647)
Supplement: S1 File — (DOCX) [file pone.0242647.s003.docx]

**Protocol: Association of polymorphisms in heat shock protein 70 genes with the susceptibility to noise-induced hearing loss (NIHL): A meta-analysis**

**Date:** March 11, 2017

**Team members:** Shimin Zong, Xue Zeng, Tianyi Liu, Fangmin Wan, Pan Luo, Hongjun xiao

**Objective:** To explore whether a relationship exists between polymorphisms in heat shock protein 70 genes and the susceptibility to noise-induced hearing loss via a meta-analysis

**Background:** NIHL has been the most recorded occupational disorder in the world. However, once NIHL occurs, few therapeutic methods would be clinically effective. Many studies in recent years have confirmed that there is a close relationship between genetic polymorphism and the occurrence of various diseases. Recent studies demonstrated that the disturbance of cellular proteostasis is critical in the development of NIHL [1]. In addition, some studies indicated a close association between several SNPs in HSP70 genes, which encode molecular chaperon HSP70, and NIHL, whereas other studies present conflictive results [2-4].

**Methods**

**1.Search strategy**

The search strategies in each database are listed as follows:

(1) **PubMed:** ((heat shock protein 70) OR (hsp70)) AND ((noise AND (hearing loss) OR (noise-induced hearing loss) OR (hearing loss, noise-induced [MeSH]) OR NIHL)

(2) **Web of Science:** ((heat shock protein 70) OR (hsp70)) AND ((noise AND (hearing loss)) OR (noise-induced hearing loss) OR (NIHL))

(3) **EMBASE (advanced search):** ((heat shock protein 70) OR (hsp70)) AND ((noise AND (hearing loss)) OR (noise-induced hearing loss) OR (NIHL))

(4) **Wanfang Chinese database:**（”热休克蛋白70”或“热应激蛋白70”）与“噪声”

**2. Inclusion criteria**

(1) Independent case-control studies investigating the relationship between the SNPs in HSP70 genes and the development of NIHL;

(2) Studies including sufficient and definite original data (the genotype frequency of each SNP in HSP70 genes in the case and control groups) in which the odds ratio (OR) with its 95% confidential interval (CI) of each genotype at every SNP site can be calculated;

(3) The data in the latest publication were used when duplicate publications were found.

**3. Type of the study to be included:** Case-control study

**4. Participants:** Workers with diagnosis of noised-induced hearing loss with healthy or resistant controls.

**5. Exposure:** Polymorphism in HSP70 genes.

**6. Main outcome:** Hearing impairment under noise conditions.

**7. Data extraction:**

Team members for data extraction: Shimin Zong, Xue Zeng, Tianyi Liu

| **Data Extraction Form** | | | | | | | | | | | |
| --- | --- | --- | --- | --- | --- | --- | --- | --- | --- | --- | --- |
| Association of polymorphisms in heat shock protein 70 genes with the susceptibility to noise-induced hearing loss (NIHL): A meta-analysis | | | | | | | | | | | |
| Title | |  | | | | | | | | | |
| Code | |  | | | | | The first author | | |  | |
| Journal | |  | | | | | Publication date | | |  | |
| Volume(Issue), Page | |  | | | | | Language | | |  | |
| Publication type | | Publication | | □ Yes □ No | | | Case-control study | | | □ Yes □ No | |
|  |  | Full text | | □ Yes □ No | | |  |  |  |  |  |
| **Population characteristics** | | | | | | | | | | | |
| Ethnicity | |  | | | | | Age range | | |  | |
| Gender | | Male | | |  | | | | Female | |  |
| Workplace | |  | | | | | | | | | |
| Noise exposure intensity | |  | | | | | | | | | |
| Hearing impairment criteria | |  | | | | | | | | | |
| Normal hearing criteria | |  | | | | | | | | | |
| Control determination | |  | | | | | | | | | |
| Number of included | | Case |  | | | | | SNP detection method | |  | |
|  |  | Control |  | | | | |  |  |  |  |
| Hearing protection: □ Yes □ No | | | | | | | | | | | |
| Genotype | | Case | | | Control | | | | OR | | 95%CI |
| AA | |  | | |  | | | |  | |  |
| AB | |  | | |  | | | |  | |  |
| BB | |  | | |  | | | |  | |  |
| Whether the control group meets HWE | | | | | | | □ Yes □ No | | | | |
| **Quality Evaluation (NOS)** | | | | | | | | | | | |
| **Selection** | 1) Is the case definition adequate | | a) yes, with independent validation * | | | | | | | |  |
|  |  |  | b) yes, eg record linkage or based on self reports | | | | | | | |  |
|  |  |  | c) no description | | | | | | | |  |
|  | 2) Representativeness of the cases | | a) consecutive or obviously representative series of cases * | | | | | | | |  |
|  |  |  | b) potential for selection biases or not stated | | | | | | | |  |
|  | 3) Selection of Controls | | a) community controls * | | | | | | | |  |
|  |  |  | b) hospital controls | | | | | | | |  |
|  |  |  | c) no description | | | | | | | |  |
|  | 4) Definition of Controls | | a) no history of disease (endpoint) * | | | | | | | |  |
|  |  |  | b) no description of source | | | | | | | |  |
| **Comparability** | Comparability of cases and controls on the basis of the design or analysis | | a) study controls for__ (Select the most important factor.) * | | | | | | | |  |
|  |  |  | b) study controls for any additional factor * (This criteria could be modified to indicate specific  control for a second important factor.) | | | | | | | |  |
| **Exposure** | 1) Ascertainment of exposure | | a) secure record (eg surgical records) * | | | | | | | |  |
|  |  |  | b) structured interview where blind to case/control status * | | | | | | | |  |
|  |  |  | c) interview not blinded to case/control status | | | | | | | |  |
|  |  |  | d) written self report or medical record only | | | | | | | |  |
|  |  |  | e) no description | | | | | | | |  |
|  | 2) Same method of ascertainment for cases and controls | | a) yes * | | | | | | | |  |
|  |  |  | b) no | | | | | | | |  |
|  | 3) Non-Response rate | | a) same rate for both groups * | | | | | | | |  |
|  |  |  | b) non respondents described | | | | | | | |  |
|  |  |  | c) rate different and no designation | | | | | | | |  |
|  | | | | | | **Total score** | | | | |  |

**Signature:**

**Date:**

**8. Genetic model:** (1) The allele (A vs. B) model,

(2) homozygote (AA vs. BB) model,

(3) heterozygote (AA vs. AB) model,

(4) dominant (AA vs. AB + BB) model,

(5) recessive (AA + AB vs. BB) model

**9. Effective size:** OR and its 95%CI

**10. Positive judgment:** There is statistical significance when the overall 95% CI do not include 1 and the p-value transformed from the Z score is less than 0.05.

**11.** **Heterogeneity evaluation：**Q test and I^2^ test.

**12. Effective model selection:** Fixed-effects model when p > 0.1 and I^2^ < 50%; otherwise the random-effects model with I^2^ ≥ 50%.

**13. Method of handling heterogeneity:** (1) Sub-group analysis based on ethnicity, study quality and the accordance with HWE in the controls. (2) Selecting random effects model to pool the effective size.

**14. Publication bias evaluation:** Begg’s funnel plots and the Egger’s test. For p<0.05 or a 95%CI that did not contain 0 in the Egger’s test, publication bias was considered present.

**15. Software:** Stata 13.1.

**Expected start and completion time:** From March 15, 2017 to June 31, 2017

**Reference:**

1. Xue Q, Li C, Chen J, Guo H, Li D, Wu X. The Protective effect of the endoplasmic reticulum stress-related factors BiP/GRP78 and CHOP/Gadd153 on noise-induced hearing loss in guinea pigs. Noise Health. 2016;18(84):247-55.

2. Chang NC, Ho CK, Lin HY, Yu ML, Chien CY, Ho KY. Association of polymorphisms of heat shock protein 70 with susceptibility to noise-induced hearing loss in the Taiwanese population. Audiol Neurootol. 2011;16(3):168-74.

3. Konings A, Van Laer L, Michel S, Pawelczyk M, Carlsson PI, Bondeson ML, et al. Variations in HSP70 genes associated with noise-induced hearing loss in two independent populations. Eur J Hum Genet. 2009;17(3):329-35.

4. Yang M, Tan H, Yang Q, Wang F, Yao H, Wei Q, et al. Association of hsp70 polymorphisms with risk of noise-induced hearing loss in Chinese automobile workers. Cell Stress Chaperones. 2006;11(3):233-9.
