# Supplementary material for: Correction: Association of polymorphisms in heat shock protein 70 genes with the susceptibility to noise-induced hearing loss: A meta-analysis
Source: PLoS One. 2020 Nov 17;15(11):e0242647. doi: 10.1371/journal.pone.0242647 (PMC7671542; doi:10.1371/journal.pone.0242647)
Supplement: S5 File — (DOCX) [file pone.0242647.s007.docx]

| **First author (year)** | **Country** | **Workplace** | **Standard of noise exposure** | **Diagnosis criteria of hearing impairment or NIHL susceptibility** | **Standard of control group** |
| --- | --- | --- | --- | --- | --- |
|  |  |  |  |  |  |
| Li (2017) | China (mainland) | Steel factory | an exposure to occupational noise higher than 80 dB(A) for more than 3 years | the average binaural hearing  threshold level (HTL) to high frequency (3000 Hz, 4000 Hz and 6000 Hz) ≥ 40 dB or the average of single hearing threshold level to linguistic frequency (500 Hz,  1000 Hz and 2000 Hz) ≥ 26 dB, | the ones who were frequency-matched by gender, age, type of work, and exposure time were also included from the remaining participators （case: control = 1:2） |
| Chang (2011) | China (Taiwan) | Factories not clearly showed | exposed to noise levels exceeding 85 dBA but limited to 90 dB time-weighted average (90 dB TWA) during working hours | threshold shift ≥ 10 dB were assigned to the noise non-susceptible (general susceptibility; GS) group* | those with a threshold shift 6 10 dB were assigned to the noise-susceptible (NS) group* |
| Konings (2009) | Sweden | 2 paper pulp mills and 1 steel factory | the majority (79%) of the subjects in the present study have been exposed to noise for 20–30 years or more. | The participators were divided into nine categories according to three age-ranges and three occupational noise exposure categories. From each category, the 10% most resistant and the 10% most sensitive persons were selected by using the hearing threshold level (HTL) at 3 kHz of the left ear. | |
|  | Poland | Different factories | an exposure to noise of at least 3 years. | The participators were divided into nine categories according to three age-ranges and three occupational noise exposure categories. From each category, the 20% most resistant and the 20% most sensitive persons were selected by using the hearing threshold level (HTL) at 4 and 6 kHz of the left ear. | |
| Yang (2006) | China (mainland) | Motor factory | an exposure to noise of at least 1 years. | Hearing threshold worse than 25 dB in either low frequency (500Hz, 1000Hz, and 2000Hz) or high frequency (4000Hz, 6000Hz, and 8000Hz) was defined as hearing loss. | Participants who do not meet the diagnostic criteria for hearing impairment |

*The noise hearing level was defined with a 4K-weighted pure-tone audiometric average (4KWPTA), which is the average of the bilateral hearing levels recorded at 3000, 6000, and two-weighted 4000 Hz.
