# Supplementary material for: Correction: Association of polymorphisms in heat shock protein 70 genes with the susceptibility to noise-induced hearing loss: A meta-analysis
Source: PLoS One. 2020 Nov 17;15(11):e0242647. doi: 10.1371/journal.pone.0242647 (PMC7671542; doi:10.1371/journal.pone.0242647)
Supplement: S6 File — (DOCX) [file pone.0242647.s008.docx]

|  |  |  |  |  |  |  |
| --- | --- | --- | --- | --- | --- | --- |
| S4 Table The results of Q test in the heterogeneity analysis | | | | | | |
| SNP | | **Ethnicity** | **Study number** | **Case/Control** | **Genetic model** | **P(Q test)** |
| rs1043618 | | **Mixed population** | 5 | 615/925 | G vs. C | 0.29 |
| (G > C) | |  |  |  | GG vs. CC | 0.35 |
|  | |  |  |  | GG vs. GC | 0.13 |
|  | |  |  |  | GG vs. GC + CC | 0.16 |
|  | |  |  |  | GG + GC vs. CC | 0.38 |
|  | | **Asian subgroup** | 3 | 406/709 | G vs. C | 0.29 |
|  | |  |  |  | GG vs. CC | 0.24 |
|  | |  |  |  | GG vs. GC | 0.11 |
|  | |  |  |  | GG vs. GC + CC | 0.17 |
|  | |  |  |  | GG + GC vs. CC | 0.16 |
|  | | **Caucasian subgroup** | 2 | 209/216 | G vs. C | 0.19 |
|  | |  |  |  | GG vs. CC | 0.31 |
|  | |  |  |  | GG vs. GC | 0.18 |
|  | |  |  |  | GG vs. GC + CC | 0.15 |
|  | |  |  |  | GG + GC vs. CC | 0.54 |
|  | | **High quality** | 4 | 525/828 | G vs. C | 0.48 |
|  | | **subgroup** |  |  | GG vs. CC | 0.42 |
|  | |  |  |  | GG vs. GC | 0.22 |
|  | |  |  |  | GG vs. GC + CC | 0.322 |
|  | |  |  |  | GG + GC vs. CC | 0.301 |
| rs2227956 | | **Mixed population** | 4 | 578/592 | A vs. G | 0.04 |
| (A > G) | |  |  |  | AA vs. GG | 0.05 |
|  | |  |  |  | AA vs. AG | 0.25 |
|  | |  |  |  | AA vs. AG + GG | 0.11 |
|  | |  |  |  | AA + AG vs. GG | 0.06 |
|  | | **Asian subgroup** | 2 | 369/377 | A vs. G | 0.86 |
|  | |  |  |  | AA vs. GG | 0.19 |
|  | |  |  |  | AA vs. AG | 0.33 |
|  | |  |  |  | AA vs. AG + GG | 0.52 |
|  | |  |  |  | AA + AG vs. GG | 0.17 |
|  | | **Caucasian subgroup** | 2 | 209/215 | **A vs. G** | 0.90 |
|  | |  |  |  | **AA vs. GG** | 0.58 |
|  | |  |  |  | **AA vs. AG** | 0.99 |
|  | |  |  |  | **AA vs. AG + GG** | 0.91 |
|  | |  |  |  | **AA + AG vs. GG** | 0.60 |
|  | | **High quality** | 3 | 470/494 | A vs. G | 0.10 |
|  | | **subgroup** |  |  | AA vs. GG | 0.09 |
|  | |  |  |  | AA vs. AG | 0.24 |
|  | |  |  |  | AA vs. AG + GG | 0.17 |
|  | |  |  |  | AA + AG vs. GG | 0.09 |
| SNP | | **Ethnicity** | **Study number** | **Case/Control** | **Genetic model** | **P(Q test)** |
| rs1061581 | | **Mixed population** | 3 | 301/315 | **A vs. G** | 0.62 |
| (A > G) | |  |  |  | **AA vs. GG** | 0.50 |
|  | |  |  |  | AA vs. AG | 0.17 |
|  | |  |  |  | **AA vs. AG + GG** | 0.25 |
|  | |  |  |  | AA + AG vs. GG | 0.38 |
|  | | **Caucasian subgroup** | 2 | 251/264 | **A vs. G** | 0.33 |
|  | | (HWE P > 0.05 |  |  | AA vs. GG | 0.49 |
|  | | subgroup) |  |  | **AA vs. AG** | 0.19 |
|  | |  |  |  | **AA vs. AG + GG** | 0.19 |
|  | |  |  |  | AA + AG vs. GG | 0.87 |
|  | | **High quality** | 2 | 107/217 | A vs. G | 0.81 |
|  | | **subgroup** |  |  | AA vs. GG | 0.26 |
|  | |  |  |  | AA vs. AG | 0.56 |
|  | |  |  |  | AA vs. AG + GG | 0.25 |
|  | |  |  |  | AA + AG vs. GG | 0.17 |
| rs2075800 | | **Asian** | 2 | 313/608 | C vs. T | 0.89 |
| (C > T) | |  |  |  | CC vs. TT | 0.84 |
|  | |  |  |  | CC vs. CT | 0.69 |
|  | |  |  |  | CC vs. CT + TT | 0.75 |
|  | |  |  |  | CC + CT vs. TT | 0.73 |
| rs2763979 | | **Asian** | 2 | 313/608 | C vs. T | 0.06 |
| (C > T) | |  |  |  | CC vs. TT | 0.18 |
|  | |  |  |  | CC vs. CT | 0.40 |
|  | |  |  |  | CC vs. CT + TT | 0.16 |
|  | |  |  |  | CC + CT vs. TT | 0.22 |
